# Spectral Decomposition of Discrepancy Kernels on the Euclidean Ball, the Special Orthogonal Group, and the Grassmannian Manifold

**DOI:** 10.1007/s00365-023-09638-0

**Published:** 2023-04-07

**Authors:** Josef Dick, Martin Ehler, Manuel Gräf, Christian Krattenthaler

**Affiliations:** 1 School of Mathematics and Statistics, University of New South Wales, Sydney, NSW 2052 Australia; 2 Department of Mathematics, University of Vienna, Oskar-Morgenstern-Platz 1, 1090 Vienna, Austria; 3 Institut für Mathematik, TU Berlin, Str. des 17. Juni 136, 10623 Berlin, Germany

**Keywords:** Discrepancy kernels, Spectral decompositions, Euclidean ball, Special orthogonal group, Grassmannian manifold, Nonequispaced fast Fourier transform, Primary 42C10 Secondary 42B10, 43A65, 43A85, 65T40

## Abstract

To numerically approximate Borel probability measures by finite atomic measures, we study the spectral decomposition of discrepancy kernels when restricted to compact subsets of $\mathbb {R}^d$. For restrictions to the Euclidean ball in odd dimensions, to the rotation group $\textrm{SO}(3)$, and to the Grassmannian manifold $\mathcal {G}_{2,4}$, we compute the kernels’ Fourier coefficients and determine their asymptotics. The $L_2$-discrepancy is then expressed in the Fourier domain that enables efficient numerical minimization based on the nonequispaced fast Fourier transform. For $\textrm{SO}(3)$, the nonequispaced fast Fourier transform is publicly available, and, for $\mathcal {G}_{2,4}$, the transform is derived here. We also provide numerical experiments for $\textrm{SO}(3)$ and $\mathcal {G}_{2,4}$.

## Introduction

Consider a Borel probability measure $\mu :\mathscr {B}(\mathbb {R}^d)\rightarrow [0,1]$ on $\mathbb {R}^d$, where $\mathscr {B}(\mathbb {R}^d)$ denotes the Borel sigma algebra on $\mathbb {R}^d$. For fixed $n\in \mathbb {N}$, we aim to allocate a suitable *n*-point set $\{x_1,\ldots ,x_n\}\subset \mathbb {R}^d$ such that the normalized atomic measure

1.1 $$\begin{aligned} \nu _n:=\frac{1}{n}\sum _{j=1}^n\delta _{x_j} \end{aligned}$$

approximates $\mu$. Here, $\delta _{x_j}:\mathscr {B}(\mathbb {R}^d)\rightarrow \{0,1\}$ denotes the point measure localized at $x_j$. To quantify the $L_2$-discrepancy between $\mu$ and $\nu _n$, select a measure $\beta$ on $\mathscr {B}(\mathbb {R}^d)$ with $\mu ,\delta _x\in L_2(\mathscr {B}(\mathbb {R}^d),\beta )$, for all $x\in \mathbb {R}^d$, and consider

1.2 $$\begin{aligned} \mathscr {D}_{\beta }(\mu ,\nu _n):=\Vert \mu -\nu _n\Vert ^2_{L_2(\mathscr {B}(\mathbb {R}^d),\beta )} =\int _{\mathscr {B}(\mathbb {R}^d)} \left| \mu (B)- \nu _n(B)\right| ^2\textrm{d} \beta (B), \end{aligned}$$

cf. [40, 42, 43], see Sect. 2 for explicit examples^1^. For fixed $n\in \mathbb {N}$, we aim to minimize $\mathscr {D}_{\beta }(\mu ,\nu _n)$ among all *n*-point sets $\{x_1,\ldots ,x_n\}\subset \mathbb {R}^d$. The present manuscript is concerned with discretizations of (1.2) that facilitate numerical minimization.

The associated *discrepancy kernel* $K_\beta :\mathbb {R}^d\times \mathbb {R}^d\rightarrow \mathbb {R}$ is defined by

1.3 $$\begin{aligned} K_\beta (x,y):= \langle \delta _x,\delta _y\rangle _{L_2(\mathscr {B}(\mathbb {R}^d),\beta )} =\int _{\mathscr {B}(\mathbb {R}^d)} \delta _x(B)\delta _y(B) \textrm{d} \beta (B), \end{aligned}$$

and we assume it is continuous. Fubini’s Theorem and $\mu (B)=\int _{\mathbb {R}^d} \delta _x(B)\textrm{d}\mu (x)$ applied to

$$\begin{aligned} \Vert \mu -\nu _n\Vert ^2_{L_2(\mathscr {B}(\mathbb {R}^d),\beta )}=\Vert \mu \Vert ^2_{L_2(\mathscr {B}(\mathbb {R}^d),\beta )}-2\langle \mu ,\nu _n\rangle _{L_2(\mathscr {B}(\mathbb {R}^d),\beta )}+\Vert \nu _n\Vert ^2_{L_2(\mathscr {B}(\mathbb {R}^d),\beta )} \end{aligned}$$

yield that (1.2) is identical to

1.4 $$\begin{aligned} \mathscr {D}_{\beta }(\mu ,\nu _n)= & {} \iint \limits _{\mathbb {R}^d\times \mathbb {R}^d} K_{\beta }(x,y)\textrm{d}\mu (x) \textrm{d}\mu (y) \nonumber \\{} & {} -2\sum _{j=1}^n\int _{\mathbb {R}^d}\frac{K_{\beta }(x,x_j)}{n}\textrm{d}\mu (x) +\sum _{i,j=1}^n \frac{K_{\beta }(x_i,x_j)}{n^2} . \end{aligned}$$

If a compact set $\mathbb {X}\subset \mathbb {R}^d$ is known in advance such that $\text {supp}(\mu )\subset \mathbb {X}$, then we shall restrict the minimization to $\{x_1,\ldots ,x_n\}\subset \mathbb {X}$, so that only the restricted kernel $K_\beta |_{\mathbb {X}\times \mathbb {X}}$ matters. By endowing $\mathbb {X}$ with a finite Borel measure $\sigma _\mathbb {X}$ having full support, Mercer’s Theorem yields an orthonormal basis $\{\phi _l\}_{l=0}^\infty$ for $L_2(\mathbb {X},\sigma _\mathbb {X})$ and coefficients $(a_l)_{l=0}^\infty$ such that the spectral decomposition

1.5 $$\begin{aligned} K_\beta |_{\mathbb {X}\times \mathbb {X}}(x,y) = \sum _{l=0}^\infty a_l \phi _l(x)\overline{\phi _l(y)},\quad x,y\in \mathbb {X}, \end{aligned}$$

holds with absolute and uniform convergence. We call $(a_l)_{l=0}^\infty$ the Fourier coefficients of the kernel $K_\beta |_{\mathbb {X}\times \mathbb {X}}$. If $\text {supp}(\mu ),\text {supp}(\nu _n)\subset \mathbb {X}$, then the Fourier expansion of the $L_2$-discrepancy (1.4) is

1.6 $$\begin{aligned}{} & {} \mathscr {D}_{\beta }(\mu ,\nu _n) = \sum _{l=0}^\infty a_l \left| \hat{\mu }_{l}-\hat{\nu }_{n,l} \right| ^2, \nonumber \\{} & {} \hat{\mu }_l{:}{=}\int _{\mathbb {X}}\overline{ \phi _l(x)}\textrm{d}\mu (x),\quad \hat{\nu }_{n,l}:=\frac{1}{n}\sum _{j=1}^n\overline{\phi _{l}(x_j)}, \end{aligned}$$

where the Fourier coefficients $\hat{\mu }_l$ and $\hat{\nu }_{n,l}$ of the measures $\mu$ and $\nu _n$, respectively, are well-defined if $a_l\ne 0$. Truncation of the discretization (1.6) enables the use of the nonequispaced fast Fourier transform, thereby offering more efficient minimization of $\mathscr {D}_\beta (\mu ,\nu _n)$, cf. [31, 33]. Thus, we aim to

A) compute $(a_l)_{l=0}^\infty$ and $(\phi _l)_{l=0}^\infty$ in the Fourier expansion (1.5) of $K_\beta |_{\mathbb {X}\times \mathbb {X}}$.

The $L_2$-discrepancy $\mathscr {D}_\beta (\mu ,\nu _n)$ also coincides with the worst case integration error

1.7 $$\begin{aligned} \mathscr {D}_\beta (\mu ,\nu _n) = \sup _{\Vert f\Vert _{\mathscr {H}_\beta (\mathbb {X})}\le 1 }\left| \int _\mathbb {X}f(x)\textrm{d}\mu (x) - \frac{1}{n}\sum _{j=1}^n f(x_j) \right| ^2 \end{aligned}$$

with respect to the reproducing kernel Hilbert space $\mathscr {H}_\beta (\mathbb {X})$ generated by $K_\beta |_{\mathbb {X}\times \mathbb {X}}$, cf. [12, 13, 30, 31]. To specify $\mathscr {H}_\beta (\mathbb {X})$, we aim to

B) identify $\mathscr {H}_\beta (\mathbb {X})$ with a classical function space.

Fourier decay properties generally quantify Sobolev smoothness. To accomplish (B), we aim to determine the asymptotics of $K_\beta |_{\mathbb {X}\times \mathbb {X}}$’s Fourier coefficients $(a_l)_{l=0}^\infty$ in (1.5).

For $\mathbb {X}=\mathbb {S}^{d-1}$ and a particular choice of $\beta$, the kernel $K_{\beta }|_{\mathbb {S}^{d-1}\times \mathbb {S}^{d-1}}$ essentially coincides with the Euclidean distance, see [12, 13]. The Fourier expansion is determined in [10], and the decay of the Fourier coefficients yields that $K_{\beta }|_{\mathbb {S}^{d-1}\times \mathbb {S}^{d-1}}$ reproduces the Sobolev space $\mathscr {H}_\beta (\mathbb {S}^{d-1})=\mathbb {H}^{\frac{d}{2}}(\mathbb {S}^{d-1})$. For the sphere and the torus, the nonequispaced fast Fourier transform is available, and both (A) and (B) are discussed in [33, 34].

This manuscript is dedicated to derive analogous results for other compact sets $\mathbb {X}$. We focus on the unit ball, the special orthogonal group, and the Grassmannian manifold,

$$\begin{aligned} \mathbb {B}^d&:=\{x\in \mathbb {R}^d:\Vert x\Vert \le 1\},\\ \textrm{SO}(d)&:=\{x\in \mathbb {R}^{d\times d} : \det (x)=1,\, x^{-1}=x^\top \},\\ \mathcal {G}_{k,d}&:=\{x\in \mathbb {R}^{d\times d} : x^\top =x,\, x^2=x,\, \textrm{trace}(x)=k\}. \end{aligned}$$

We achieve goal (A) for $\mathbb {X}=\mathbb {B}^d$ with odd *d*. Both goals, (A) and (B), are achieved for $\textrm{SO}(3)$ and $\mathcal {G}_{2,4}$. We also provide numerical experiments. For $\textrm{SO}(3)$, the computations are based on the nonequispaced fast Fourier transform designed in [32, 44]. For $\mathcal {G}_{2,4}$, we derive the nonequispaced fast Fourier transform by parametrization via the double covering $\mathbb {S}^2\times \mathbb {S}^2$ and developing the respective transform there.

For $\textrm{SO}(d)$ and $\mathcal {G}_{k,d}$ with fixed *k* and *d*, in principle, one could still be able to compute the Fourier expansion in goal (A). However, one may be faced with rather complicated expressions. In our present computations for $\textrm{SO}(3)$ and $\mathcal {G}_{2,4}$, the structural relations to the unit sphere enabled the use of Chebychev and Legendre polynomials, which reduced the complexity. Nonetheless, we do accomplish (B) for the general cases $\textrm{SO}(d)$ and $\mathcal {G}_{k,d}$.

## Two Introductory Examples

We first present a well-known elementary example on the interval [0, *s*], for which both aims A) and B) are achieved. Second, to support our perspective on discrepancy, we prove that the so-called Askey function is a discrepancy kernel of the form (1.3).

### The Brownian Motion Kernel on [0, *s*]

Let $\textrm{d}r$ be the Lebesgue measure on $[0,\infty )$. The mapping $h:[0,\infty )\rightarrow \mathscr {B}(\mathbb {R})$ defined by $r\mapsto [r,\infty )$ induces the pushforward measure $\beta :=h_*(\textrm{d}r)$ that induces the discrepancy

$$\begin{aligned} \mathscr {D}_\beta (\mu ,\nu _n)= \int _{0}^\infty |\mu ([r,\infty ))-\nu _n([r,\infty ))|^2 \textrm{d}r. \end{aligned}$$

The associated discrepancy kernel $K_\beta :\mathbb {R}\times \mathbb {R}\rightarrow \mathbb {R}$ is^2^

$$\begin{aligned} K_\beta (x,y) = \int _0^\infty \delta _x([r,\infty ))\delta _y([r,\infty )) \textrm{d}r =\min (x,y)_+, \end{aligned}$$

so that $\mathscr {D}_\beta (\delta _x,\delta _y)=|x-y|$ for $x,y\in [0,\infty )$. The restriction of the kernel $K_\beta$ to $[0,s]\times [0,s]$ has the Fourier expansion

$$\begin{aligned} K_\beta (x,y)&=\sum _{\begin{array}{c} m\in \mathbb {N}\\ m \text { odd} \end{array}} \frac{4s^2}{m^2\pi ^2}\cdot \frac{\sin (\frac{\pi }{2s} mx)}{\sqrt{\frac{s}{2}}}\cdot \frac{\sin (\frac{\pi }{2s} my)}{\sqrt{\frac{s}{2}}},\quad x,y\in [0,s], \end{aligned}$$

with respect to the Lebesgue measure $\sigma _{[0,s]}$ on [0, *s*]. The reproducing kernel Hilbert space is

$$\begin{aligned} \mathscr {H}_{\beta }([0,s])=\{f:[0,s]\rightarrow \mathbb {C}\;:\; f \text { is absolutely continuous, } f(0)=0,\; f'\in L_2([0,s])\}, \end{aligned}$$

where the inner product between *f* and *g* is given by $\langle f',g'\rangle _{L_2([0,s])}$, cf. [3, 22] and [43, Section 9.5.5]. Note that $K_\beta |_{[0,1]\times [0,1]}$ is often called the Brownian motion kernel and $\mathscr {H}_{\beta }([0,s])$ is continuously embedded into the Sobolev space $\mathbb {H}^1([0,s])$.

### Askey’s Function and Its Restrictions

Many positive definite kernels in the literature are of the form (1.3) and, hence, are discrepancy kernels. For odd *d*, Askey’s kernel function $(x,y)\mapsto (1-\Vert x-y\Vert )^{\frac{d+1}{2}}_+$ is positive definite, cf. [29]. In the following, we shall check that it is of the form (1.3).

Denote the Euclidean ball of radius *s* centered at $z\in \mathbb {R}^d$ by

$$\begin{aligned} \mathbb {B}^d_{s}(z):=\{x\in \mathbb {R}^d:\Vert x-z\Vert \le {s}\}, \end{aligned}$$

with the conventions $\mathbb {B}^d_{s}:=\mathbb {B}^d_{s}(0)$ and $\mathbb {B}^d:=\mathbb {B}^d_1$. Fix $r>0$ and consider the discrepancy

2.1 $$\begin{aligned} \mathscr {D}_{d,r}(\mu ,\nu _n):=\frac{1}{\textrm{vol}(\mathbb {B}^d_{\frac{r}{2}})} \int _{\mathbb {R}^d} \left| \mu (\mathbb {B}^d_{\frac{r}{2}}(z))- \nu _n(\mathbb {B}^d_{\frac{r}{2}}(z))\right| ^2\textrm{d} z, \end{aligned}$$

where $\textrm{vol}(\mathbb {B}^d_{\frac{r}{2}})=\frac{\pi ^{d/2}}{\Gamma (\frac{d}{2}+1)}(\frac{r}{2})^d$. The associated discrepancy kernel is

2.2 $$\begin{aligned} K_{d,r}(x,y)=\frac{1}{\textrm{vol}(\mathbb {B}^d_{\frac{r}{2}})}\int _{\mathbb {R}^d} \delta _x(\mathbb {B}^d_{\frac{r}{2}}(z))\delta _y(\mathbb {B}^d_{\frac{r}{2}}(z)) \textrm{d} z.\nonumber \\ \end{aligned}$$

In order to additionally integrate over *r*, recall the (generalized) hypergeometric functionswhere $f_1,\ldots ,f_k,g_1,\ldots ,g_l,z\in \mathbb {R}$ and $(f)_n:=f\cdot (f+1)\cdots (f+n-1)$ denotes the Pochhammer symbol with $(f)_0:=1$. We consider $G_d:[0,\infty )\rightarrow \mathbb {R}$ given bySince *d* is odd, either $\frac{d+1}{4}$ or $\frac{d-1}{4}$ is a natural number, so that the series terminates and $G_d$ is a polynomial in $r^{2}$ on [0, 1]. By integration with respect to $G_d$, we obtain the $L_2$-discrepancy and the associated discrepancy kernel

$$\begin{aligned} \mathscr {D}_{d}(\mu ,\nu _n):= \int _0^\infty \!\!\mathscr {D}_{d,r}(\mu ,\nu _n)\textrm{d} G_d(r),\quad \text { and }\quad K_{d}(x,y)= \int _0^\infty \!K_{d,r}(x,y) \textrm{d} G_d(r), \end{aligned}$$

respectively. It turns out that $K_d$ coincides with Askey’s function.

#### Theorem 2.1

Let *d* be odd. The discrepancy kernel $K_d$ satisfies

2.3 $$\begin{aligned} K_d(x,y)=(1-\Vert x-y\Vert )^{\frac{d+1}{2}}_+,\quad x,y\in \mathbb {R}^d. \end{aligned}$$

The proof is presented in Appendix A. Provided that $d\ge 3$, Askey’s kernel function reproduces the Sobolev space $\mathbb {H}^{\frac{d+1}{2}}(\mathbb {R}^d)$ with an equivalent norm, see [49].

## The Distance Kernel on $\mathbb {S}^{d-1}$

This section is dedicated to recall results on discrepancy kernels on the sphere $\mathbb {S}^{d-1}\subset \mathbb {R}^d$, for $d\ge 2$, from [12, 13, 31, 46] that shall guide our subsequent investigations.

Denote the geodesic ball of radius *r* centered at $z\in \mathbb {S}^{d-1}$ by

$$\begin{aligned} B^{\mathbb {S}^{d-1}}_r(z):= \{x\in \mathbb {S}^{d-1} : \textrm{dist}_{\mathbb {S}^{d-1}}(x,z)\le r\}, \end{aligned}$$

where $\textrm{dist}_{\mathbb {S}^{d-1}}(x,z)=\arccos (\langle x,z\rangle )$ is the geodesic distance on $\mathbb {S}^{d-1}$. We define

$$\begin{aligned} h:[0,\pi ]\times \mathbb {S}^{d-1}\rightarrow \mathscr {B}(\mathbb {R}^d),\qquad (r,z)\mapsto B^{\mathbb {S}^{d-1}}_r(z) \end{aligned}$$

and endow $[0,\pi ]$ with the weighted Lebesgue measure $\sin (r){\mathrm d}r$, whereas $\mathbb {S}^{d-1}$ carries the normalized, orthogonal invariant surface measure $\sigma _{\mathbb {S}^{d-1}}$. The push-forward $\beta _d:=h_*(\sin (r){\mathrm d}r \otimes \sigma _{\mathbb {S}^{d-1}})$ is a measure on $\mathscr {B}(\mathbb {R}^d)$, so that the associated $L_2$-discrepancy is

$$\begin{aligned} \mathscr {D}_{\beta _d}(\mu ,\nu _n)= \int _{0}^\pi \int _{\mathbb {S}^{d-1}} |\mu (B^{\mathbb {S}^{d-1}}_r(z)) -\nu _n(B^{\mathbb {S}^{d-1}}_r(z))|^2 \textrm{d}\sigma _{\mathbb {S}^{d-1}}(z)\sin (r)\textrm{d}r. \end{aligned}$$

The associated discrepancy kernel is

3.1 $$\begin{aligned} K_{\beta _d}(x,y)=\int _{0}^\pi \int _{\mathbb {S}^{d-1}}\delta _x(B^{\mathbb {S}^{d-1}}_r(z))\delta _y(B^{\mathbb {S}^{d-1}}_r(z)) \textrm{d}\sigma _{\mathbb {S}^{d-1}}(z)\sin (r)\textrm{d}r,\quad x,y\in \mathbb {R}^{d}.\nonumber \\ \end{aligned}$$

According to [12, 13, 31], see also [2], $K_{\beta _d}$ satisfies

3.2 $$\begin{aligned} K_{\beta _d}(x,y)=1-\frac{\Gamma (\frac{d}{2})}{2\sqrt{\pi }\Gamma (\frac{d+1}{2})}\Vert x-y\Vert ,\qquad x,y\in \mathbb {S}^{d-1}. \end{aligned}$$

If either *x* or *y* is not contained in $\mathbb {S}^{d-1}$, then $K_{\beta _d}(x,y)=0$.

Choose $\sigma _\mathbb {X}:=\sigma _{\mathbb {S}^{d-1}}$ for the decomposition (1.5) and let $\{Y^m_{{l}} : l=1,\ldots ,Z(d,m)\}\subset L_2(\mathbb {S}^{d-1},\sigma _{\mathbb {S}^{d-1}})$ denote the set of orthonormal spherical harmonics of degree *m* on $\mathbb {S}^{d-1}$, where $$ Z(d,m)=\frac{2m+d-2}{d-2}\left( {\begin{array}{c}m+d-3\\ m\end{array}}\right) . $$ For $\tau >(d-1)/2$, the Sobolev space $\mathbb {H}^\tau (\mathbb {S}^{d-1})$ is the reproducing kernel Hilbert space associated with the reproducing kernel

3.3 $$\begin{aligned} (x,y)\mapsto \sum _{m=0}^\infty (1+m(m+d-2))^{-\tau }\sum _{l=1}^{Z(d,m)}Y^m_{l}(x)\overline{Y^m_{l}(y)},\quad x,y\in \mathbb {S}^{d-1}. \end{aligned}$$

The coefficients in the Fourier expansion

$$\begin{aligned} 1-\frac{\Gamma (\frac{d}{2})}{2\sqrt{\pi }\Gamma (\frac{d+1}{2})}\Vert x-y\Vert = \sum _{m=0}^\infty c_m \sum _{l=1}^{Z(d,m)}Y^m_{l}(x)\overline{Y^m_{l}(y)},\quad x,y\in \mathbb {S}^{d-1}, \end{aligned}$$

satisfy $|c_m|\sim m^{-d}$, cf. [12]. This is the same asymptotics as the coefficients in (3.3) for $\tau =d/2$. Therefore, $K_{\beta _d}|_{\mathbb {S}^{d-1}\times \mathbb {S}^{d-1}}$ reproduces the Sobolev space $\mathscr {H}_{\beta _d}(\mathbb {S}^{d-1})=\mathbb {H}^{\frac{d}{2}}(\mathbb {S}^{d-1})$ with an equivalent norm^3^.

In order to determine the Fourier coefficients of kernels on the sphere that are polynomial in $\Vert x-y\Vert$, such as $K_{\beta _d}|_{\mathbb {S}^{d-1}\times \mathbb {S}^{d-1}}$, we require the Fourier coefficients of the monomial terms $\Vert x-y\Vert ^p$. For any $p\in \mathbb {N}$, the Fourier expansion

3.4 $$\begin{aligned} 2^{-\frac{p}{2}}\Vert x-y\Vert ^p = \sum _{m=0}^\infty a_m(p,\mathbb {S}^{d-1}) \sum _{l=1}^{Z(d,m)}Y^m_{l}(x) \overline{Y^m_{l}(y)},\quad x,y\in \mathbb {S}^{d-1}, \end{aligned}$$

holds with coefficients determined by

3.5 $$\begin{aligned} a_m(p,\mathbb {S}^{d-1}):=\frac{1}{Z(d,m)}\iint \limits _{\mathbb {S}^{d-1}\times \mathbb {S}^{d-1}} 2^{-\frac{p}{2}} \Vert x-y\Vert ^p \sum _{l=1}^{Z(d,m)}\overline{Y^m_{l}(x)}Y^m_{l}(y) \textrm{d}\sigma _{\mathbb {S}^{d-1}}(x)\textrm{d}\sigma _{\mathbb {S}^{d-1}}(y).\nonumber \\ \end{aligned}$$

Note that (3.5) is well-defined for the entire range $p>-(d-1)$ and *p* is not required to be an integer. For $p>0$, the following proposition is essentially due to [10], see also [12, 14]. Simple continuation arguments cover the full range of *p*, and the asymptotics $\frac{\Gamma (-\frac{p}{2}+m)}{\Gamma (\frac{p}{2}+d-1+m)}=m^{-(p+d-1)}(1+o(1))$ are standard.

### Proposition 3.1

( [10]) Suppose $d\ge 2$. For any $p >-(d-1)$, we have

3.6 $$\begin{aligned} a_m(p,\mathbb {S}^{d-1})&=\frac{2^{d} \Gamma (\frac{d}{2})}{4\sqrt{\pi }}\cdot \frac{2^{p/2} \Gamma (\frac{d}{2}+\frac{p}{2}-\frac{1}{2})}{\Gamma (-\frac{p}{2})}\cdot \frac{\Gamma (-\frac{p}{2}+m)}{\Gamma (\frac{p}{2}+d-1+m)}. \end{aligned}$$

In particular, if $p\not \in 2\mathbb {N}$, then

3.7 $$\begin{aligned} |a_m(p,\mathbb {S}^{d-1})|&=\left| \frac{2^{d} \Gamma (\frac{d}{2})}{4\sqrt{\pi }} \cdot \frac{2^{p/2} \Gamma (\frac{d}{2}+\frac{p}{2}-\frac{1}{2})}{\Gamma (-\frac{p}{2})}\right| m^{-(p+d-1)}(1+o(1)), \end{aligned}$$

and the series (3.4) terminates if $p\in 2\mathbb {N}$.

For $p\in 2\mathbb {N}$, the term $\Gamma (-\frac{p}{2})$ is not well-defined and (3.6) is to be understood with the convention $\frac{\Gamma (-\frac{p}{2}+m)}{\Gamma (-\frac{p}{2})}=(-\frac{p}{2})_m$. Hence, we observe $a_m(p,\mathbb {S}^{d-1}) = 0$ for all $m > p/2$ if $p\in 2\mathbb {N}$.

It is noteworthy that the kernel $K_{d,r}$ in (2.2) for $d=3$ is a discrepancy kernel that does not generate a Sobolev space on $\mathbb {R}^d$ but its restriction does. The proof of the following proposition is presented in Appendix B.

### Proposition 3.2

Let $r\ge 1$. The reproducing kernel Hilbert space of $K_{3,r}$, given by (2.2) with $d=3$, is continuously embedded into $\mathbb {H}^{2}(\mathbb {R}^3)$, but the reverse embedding does not hold. In contrast, $K_{3,r}|_{\mathbb {S}^{2}\times \mathbb {S}^{2}}$ reproduces $\mathbb {H}^{\frac{3}{2}}(\mathbb {S}^{2})$ with an equivalent norm.

To provide numerical examples for $d=3$, Proposition 3.1 provides the coefficients $(a_m)_{m=0}^\infty$ in the kernel expansion of $K_{\beta _{3}}$,

$$\begin{aligned} 1-\frac{1}{4}\Vert x-y\Vert = \sum _{m=0}^\infty a_m \sum _{l=1}^{2m+1}Y^m_{l}(x)\overline{Y^m_{l}(y)},\quad x,y\in \mathbb {S}^2. \end{aligned}$$

For $\text {supp}(\mu ),\text {supp}(\nu _n)\subset \mathbb {S}^2$, the $L_2$-discrepancy (1.6) for $K_{\beta _{3}}$ with $\mathbb {X}=\mathbb {S}^2$ becomes

3.8 $$\begin{aligned} \mathscr {D}_{\beta _{3}}(\mu ,\nu _n) = \sum _{m=0}^\infty a_m \sum _{l=1}^{2m+1} \left| \hat{\mu }^m_{l}- \frac{1}{n}\sum _{j=1}^n\overline{Y^m_{l}(x_j)}\right| ^2, \end{aligned}$$

where $\hat{\mu }^m_{l}$ denotes the Fourier coefficient of $\mu$ with respect to $Y^m_{l}$, cf. (1.6). By truncating this series, the nonequispaced fast Fourier transform on $\mathbb {S}^2$, cf. [33, 39, 41], enables efficient minimization of

**Fig. 1:**
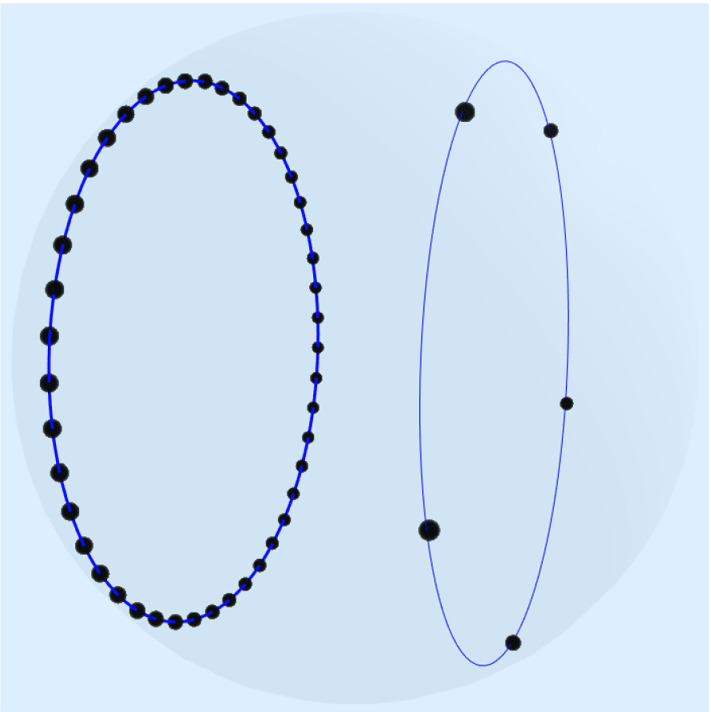
The target measure $\mu$ is supported on two circles on the sphere $\mathbb {S}^2$ with weight ratio 9/1. Numerical minimization of (3.9) splits 50 points into 45 points equally distributed on one and 5 points on the other circle

3.9 $$\begin{aligned} \sum _{m=0}^M a_m \sum _{l=1}^{2m+1} \left| \hat{\mu }^m_{l}- \frac{1}{n}\sum _{j=1}^n\overline{Y^m_{l}(x_j)}\right| ^2 \end{aligned}$$

among all *n*-point sets $\{x_1,\ldots ,x_n\}\subset \mathbb {S}^2$ for fixed *n*. We are most interested in $n\gg M$. See Figure 1 for a numerical experiment with $M=8$ and $n=50$.

## Discrepancy Kernels on Compact Sets

Here we discuss discrepancy kernels that extend the kernels of the previous section in a natural way. For $d\ge 1$, let us define the half-space

$$\begin{aligned} \Omega ^d_r(z):=\{x\in \mathbb {R}^d : \langle z,x\rangle \ge r \}\in \mathscr {B}(\mathbb {R}^d),\quad z\in \mathbb {S}^{d-1},\quad r\in \mathbb {R}. \end{aligned}$$

For fixed $s>0$, we consider the mapping $h:[-s,s]\times \mathbb {S}^{d-1}\rightarrow \mathscr {B}(\mathbb {R}^d)$ defined by $(r,z)\mapsto \Omega ^d_r(z)$ and endow $[-s,s]$ with the Lebesgue measure ${\mathrm d}r$. The push-forward measure $\beta _{d,s}:=h_*({\mathrm d}r\otimes \sigma _{\mathbb {S}^{d-1}})$ leads to the associated $L_2$-discrepancy

$$\begin{aligned} \mathscr {D}_{\beta _{d,s}}(\mu ,\nu _n)=\int _{-s}^{s} \int _{\mathbb {S}^{d-1}} \left| \mu (\Omega ^d_r(z)) - \nu _n(\Omega ^d_r(z))\right| ^2\textrm{d} \sigma _{\mathbb {S}^{d-1}}(z) \textrm{d} r. \end{aligned}$$

The associated *discrepancy kernel* is

4.1 $$\begin{aligned} K_{\beta _{d,s}}(x,y)=\int _{-s}^{s} \int _{\mathbb {S}^{d-1}} \delta _x(\Omega ^d_r(z))\delta _y(\Omega ^d_r(z)) \textrm{d} \sigma _{\mathbb {S}^{d-1}}(z) \textrm{d} r,\qquad x,y\in \mathbb {R}^d. \end{aligned}$$

Since $B^{\mathbb {S}^{d-1}}_r(z) = \Omega ^d_{\cos (r)}(z)\cap \mathbb {S}^{d-1}$, for $r\in [0,\pi ]$ and $z\in \mathbb {S}^{d-1}$, we deduce

$$\begin{aligned} K_{\beta _d}|_{\mathbb {S}^{d-1}\times \mathbb {S}^{d-1}}=K_{\beta _{d,1}}|_{\mathbb {S}^{d-1}\times \mathbb {S}^{d-1}},\qquad d\ge 2, \end{aligned}$$

with $K_{\beta _d}$ as in (3.1). In contrast to $K_{\beta _d}$, the kernel $K_{\beta _{d,s}}$ is not identically zero outside of $\mathbb {S}^{d-1}\times \mathbb {S}^{d-1}$ and makes also sense for $d=1$.

### Example 4.1

For $d=1$, we have $\mathbb {S}^0=\{\pm 1\}$, so that the half-spaces are $\Omega ^1_r(1)=[r,\infty )$ and $\Omega ^1_r(-1)=(-\infty ,-r]$. Direct calculation of (4.1) yields

$$\begin{aligned} K_{\beta _{1,s}}(x,y)= {\left\{ \begin{array}{ll} s-\frac{1}{2}|x-y|,&{} |x|,|y|\le s,\\ \frac{s}{2}+\frac{xy}{2|y|},&{} |x|\le s\le |y|,\\ s\textrm{H}(xy), &{} s\le |x|,|y|, \end{array}\right. } \end{aligned}$$

where $\textrm{H}$ is the Heaviside step function.

### Proposition 4.2

The Fourier expansion of the kernel $K_{\beta _{1,s}}|_{[-s,s]\times [-s,s]}$ with respect to the Lebesgue measure $\sigma _{[-s,s]}$ on $[-s,s]$ is

$$\begin{aligned} K_{\beta _{1,s}}(x,y)= & {} \sum _{\begin{array}{c} m\in \mathbb {N}\\ m \text { odd} \end{array}} \frac{4s^2}{m^2\pi ^2}\cdot \frac{1}{s} \cdot \sin (\frac{\pi }{2s}mx)\sin (\frac{\pi }{2s}my) \\{} & {} + \sum _{\{u>0 \,:\,\tan (u)=\frac{1}{u}\} } \frac{s^2}{u^2}\cdot \frac{1}{s(\sin (u)^2+1)} \cdot \cos (\frac{u}{s}x)\cos (\frac{u}{s}y),\quad x,y\in [-s,s]. \end{aligned}$$

Its reproducing kernel Hilbert space is

$$\begin{aligned} \mathscr {H}_{K_{\beta _{1,s}}}([-s,s])=\{f:[-s,s]\rightarrow \mathbb {C}\;:\; f \text { is absolutely continuous, } f'\in L_2([-s,s])\}, \end{aligned}$$

where the inner product between *f* and *g* is given by

$$\begin{aligned} \frac{1}{2s}\big (f(-s)+f(s)\big )\big (\overline{g(-s)+g(s)} \big ) +\langle f',g'\rangle _{L_2([-s,s])}. \end{aligned}$$

Note that $\mathscr {H}_{K_{\beta _{1,s}}}([-s,s])$ is continuously embedded into $\mathbb {H}^1([-s,s])$. The proof of Proposition 4.2 is presented in Appendix C. It uses that, up to a constant, $K_{\beta _{1,s}}|_{[-s,s]\times [-s,s]}$ is the Green’s function of the 1-dimensional harmonic equation $\Delta u = f$ on $[-s,s]$ with the boundary conditions $u'(s) = - u'(-s)$ and $u(s) + u(-s) = -2u'(s)$.

It turns out that $K_{\beta _{d,s}}$ has a simple form on $\mathbb {B}^d_{s}\times \mathbb {B}^d_s$.

### Theorem 4.3

For $d\ge 1$, the discrepancy kernel $K_{\beta _{d,s}}$ satisfies

4.2 $$\begin{aligned} K_{\beta _{d,s}}(x,y) = s-\frac{\Gamma (\frac{d}{2})}{2\sqrt{\pi }\Gamma (\frac{d+1}{2})}\Vert x-y\Vert ,\qquad x,y\in \mathbb {B}^d_{s}. \end{aligned}$$

The identity (4.2) for $x,y\in \mathbb {S}^{d-1}$ with $s=1$ has been established in [13], see also (3.2). Essentially, the same proof still works for the more general situation. Theorem 4.3 provides a simple form of $K_{\beta _{d,s}}|_{\mathbb {X}\times \mathbb {X}}$ with $\mathbb {X}= \mathbb {B}^d_{s}$, which may facilitate further computations. An immediate consequence is $\mathscr {D}_{\beta _{d,s}}(\delta _x,\delta _y)=\frac{\Gamma (\frac{d}{2})}{\sqrt{\pi }\Gamma (\frac{d+1}{2})}\Vert x-y\Vert$, for $x,y\in \mathbb {B}^d_{s}$.

## The Euclidean Ball $\mathbb {B}^d$

This section is dedicated to derive the Fourier expansion of the discrepancy kernel $K_{\beta _{d,1}}$ in (4.1) on $\mathbb {B}^d$. Proposition 4.2 has covered $d=1$, and we now derive the spectral decomposition of

$$\begin{aligned} K^{d,p}:\mathbb {B}^d\times \mathbb {B}^d \rightarrow \mathbb {R},\qquad (x,y)\mapsto \Vert x-y\Vert ^p, \end{aligned}$$

for all odd *d* with odd $p>1-d$ with respect to the Lebesgue measure $\sigma _{\mathbb {B}^d}$ on $\mathbb {B}^d$. The case $d=3$ with $p=-1$ is discussed in [37].

Let $\{\mathcal {C}^{\alpha }_m: m\in \mathbb {N},\; \alpha >-1/2\}$ denote the family of Gegenbauer polynomials with the standard normalization

$$\begin{aligned} \mathcal {C}^{\alpha }_m(1) =\left( {\begin{array}{c}m+2\alpha -1\\ m\end{array}}\right) = \frac{\Gamma (m+2\alpha )}{\Gamma (2\alpha )\Gamma (m+1)},\quad \alpha \ne 0. \end{aligned}$$

By $\alpha =\frac{d}{2}-1$, the addition theorem for spherical harmonics yields

5.1 $$\begin{aligned} \sum _{l=1}^{Z(d,m)} Y^m_{l}(x)\overline{Y^m_{l}(y)} = \tfrac{2m+d-2}{d-2} \mathcal {C}^{\frac{d}{2}-1}_m(\langle x,y\rangle ),\quad x,y\in \mathbb {S}^{d-1}. \end{aligned}$$

For $m\in \mathbb {N}$, let us define the kernels $\mathcal {K}^{d,p}_m:[0,1]\times [0,1]\rightarrow \mathbb {R}$,5.2For $d\ge 3$ and arbitrary real $p>1-d$, we deduce from [18] that

5.3 $$\begin{aligned} \Vert x-y\Vert ^p = \sum _{m=0}^\infty \mathcal {K}^{d,p}_m(\Vert x\Vert ,\Vert y\Vert ) \,\mathcal {C}^{\frac{d}{2}-1}_m \big (\big \langle \tfrac{x}{\Vert x\Vert },\tfrac{y}{\Vert y\Vert }\big \rangle \big ), \quad x,y\in \mathbb {B}^d,\; (x\ne y \text { if } p < 0).\nonumber \\ \end{aligned}$$

For $x=0$ or $y=0$, the right-hand side of (5.3) is well-defined by analytic continuation.

Using the addition formula (5.1) we obtain

$$\begin{aligned} K^{d,p}(x,y) = \sum _{m=0}^\infty \tfrac{d-2}{2m+d-2} \mathcal {K}^{d,p}_m(\Vert x\Vert ,\Vert y\Vert ) \sum _{l=1}^{Z(d,m)}Y^{m}_{l}(\tfrac{x}{\Vert x\Vert }) Y^{m}_{l}(\tfrac{y}{\Vert y\Vert }),\quad x,y\in \mathbb {B}^d. \end{aligned}$$

The Fourier expansion of ${\mathcal {K}}_{m}^{d,p}$ with respect to the measure $r^{d-1} \mathrm dr$ satisfies

5.4 $$\begin{aligned}{} & {} {\mathcal {K}}_{m}^{d,p}(r,s) = \sum _{j=1}^{\infty } \lambda _{m,j}^{d,p} \varphi _{m,j}^{d,p}(r) \varphi _{m,j}^{d,p}(s),\nonumber \\{} & {} \quad \int _{0}^{1} {\mathcal {K}}_{m}^{d,p}(r,s) \varphi _{m,j}^{d,p}(r)r^{d-1} \mathrm d r = \lambda _{m,j}^{d,p} \varphi ^{d,p}_{m,j}(s), \end{aligned}$$

where $\int _{0}^{1} |\varphi _{m,j}^{d,p}(r)|^{2} r^{d-1}\mathrm dr = 1$. We consider

$$\begin{aligned} \varphi _{m',j',l'}^{d,p}(x):= \varphi _{m',j'}^{d,p}(\Vert x\Vert ) Y^{m'}_{l'}(\tfrac{x}{\Vert x\Vert }), \quad x \in {\mathbb {B}}^{d}, \end{aligned}$$

as well as the integral operator in Mercer’s theorem induced by $K^{d,p}$,

5.5 $$\begin{aligned} T^{d,p} : L_2(\mathbb {B}^d) \rightarrow \mathcal {C}(\mathbb {B}^d), \quad f\mapsto \int _{\mathbb {B}^d} K^{d,p}(\cdot ,y)f(y)\textrm{d} y, \end{aligned}$$

so that direct computations yield

5.6 $$\begin{aligned} T^{d,p} \varphi _{m',j',l'}^{d,p}(x) = \lambda _{m',j'}^{d,p} \tfrac{(d-2) \textrm{vol}({\mathbb {S}}^{d-1})}{2m'+d-2} \varphi _{m',j',l'}^{d,p}(x) \end{aligned}$$

with the scaling $\int _{{\mathbb {B}}^{d}}\left| \varphi _{m',j',l'}^{d,p}(x)\right| ^{2} \mathrm d x = \textrm{vol}({\mathbb {S}}^{d-1})$. This leads to the Fourier expansion

5.7 $$\begin{aligned} K^{d,p} (x,y)&= \sum _{m=0}^\infty \sum _{j=1}^\infty \lambda _{m,j}^{d,p} \tfrac{(d-2)\textrm{vol} ({\mathbb {S}}^{d-1})}{2m+d-2} \sum _{l=1}^{Z(d,m)}\frac{\varphi ^{d,p}_{m,j,l}(x) }{\sqrt{\textrm{vol}({\mathbb {S}}^{d-1})} } \frac{\varphi ^{d,p}_{m,j,l}(y)}{\sqrt{\textrm{vol}({\mathbb {S}}^{d-1})}} ,\quad x,y\in \mathbb {B}^d. \end{aligned}$$

Thus, the original problem is reduced to the spectral decomposition of the sequence of kernels $\mathcal {K}^{d,p}_m$, for $m\in \mathbb {N}$. The kernel $\mathcal {K}^{d,p}_m$ induces the integral operator

5.8 $$\begin{aligned} T_m^{d,p} : L_2([0,1],r^{d-1}\textrm{d}r) \rightarrow \mathcal {C}([0,1]), \quad f\mapsto \int _0^1 \mathcal {K}^{d,p}_m(\cdot ,r)f(r)r^{d-1}\textrm{d}r, \end{aligned}$$

with eigenvalues $\lambda ^{d,p}_{m,j}$ and eigenfunctions $\varphi ^{d,p}_{m,j}$. We now specify these eigenvalues and eigenfunctions. In the following theorem, $J_{\nu }$ denotes the Bessel function of the first kind of order $\nu$ and $\zeta _{k}:=\textrm{e}^{2\pi \textrm{i}/k}$ is the *k*-th root of unity.

### Theorem 5.1

Suppose that both $d\ge 3$ and $p>1-d$ are odd and let $m\in \mathbb {N}$. Then the following holds:

- Any eigenvalue $\lambda \ne 0$ of $T_m^{d,p}$ is in a one-to-one correspondence with
  5.9 $$\begin{aligned} \omega = \big |\lambda ^{-1}2^{d+p-2}(d+2m-2)(-\tfrac{p}{2})_{\frac{d+p}{2}-1}(\tfrac{d+p}{2}-1)!\big |^{\frac{1}{d+p}} \end{aligned}$$
  with $\omega$ satisfying $\det (A(\omega ))=0$, where
  5.10 $$\begin{aligned} A(\omega ) = {\left\{ \begin{array}{ll} \left( \zeta _{d+p}^{-i\ell } J_{m+\frac{d}{2}-i-1}(\zeta _{d+p}^{\ell }\omega ) \right) _{i=1,\,\ell =0}^{\frac{d+p}{2},\,\frac{d+p}{2}-1}, &{} (-\tfrac{p}{2} )_{\frac{d+p}{2}-1} \lambda > 0,\\ \left( \zeta _{2(d+p)}^{-i(2\ell +1)}J_{m+\frac{d}{2}-i-1}(\zeta _{2(d+p)}^{2\ell +1}\omega ) \right) _{i=1,\,\ell =0}^{\frac{d+p}{2},\,\frac{d+p}{2}-1}, &{} (-\tfrac{p}{2} )_{\frac{d+p}{2}-1} \lambda < 0. \end{array}\right. }\nonumber \\ \end{aligned}$$
- The eigenfunctions are exactly
  $$\begin{aligned} r\mapsto {\left\{ \begin{array}{ll} \sum _{\ell =1}^{\frac{d+p}{2}} c_\ell \,r^{1-\frac{d}{2}}J_{m+\frac{d}{2}-1}(\zeta ^{\ell -1}_{d+p}\omega r ), &{} (-\tfrac{p}{2} )_{\frac{d+p}{2}-1} \lambda > 0,\\ \sum _{\ell =1}^{\frac{d+p}{2}} c_\ell \,r^{1-\frac{d}{2}}J_{m+\frac{d}{2}-1}(\zeta ^{2\ell -1}_{2(d+p)}\omega r ), &{} (-\tfrac{p}{2} )_{\frac{d+p}{2}-1} \lambda < 0, \end{array}\right. } \end{aligned}$$
  where $c\in \mathbb {R}^{\frac{d+p}{2}}$ is in the nullspace of $A(\omega )$.

### Remark 5.2

Computer experiments seem to indicate that the nullspace of $A(\omega )$ is one-dimensional if $\det (A(\omega ))=0$. In that case, the function

5.11 $$\begin{aligned} r\mapsto {\left\{ \begin{array}{ll} \sum _{\ell =1}^{\frac{d+p}{2}} (-1)^{\ell } A_{[1,\ell ]}(\omega ) \,r^{1-\frac{d}{2}}J_{m+\frac{d}{2}-1} (\zeta ^{\ell -1}_{d+p}\omega r ),&{} (-\tfrac{p}{2} )_{\frac{d+p}{2}-1} \lambda > 0,\\ \sum _{\ell =1}^{\frac{d+p}{2}} (-1)^{\ell } A_{[1,\ell ]}(\omega ) \,r^{1-\frac{d}{2}}J_{m+\frac{d}{2}-1} (\zeta ^{2\ell -1}_{2(d+p)}\omega r ),&{} (-\tfrac{p}{2} )_{\frac{d+p}{2}-1} \lambda < 0, \end{array}\right. }\nonumber \\ \end{aligned}$$

where $A_{[1,\ell ]}(\omega )$ denotes the $(1,\ell )$ minor of $A(\omega )$, spans the eigenspace associated with $\lambda$.

Appendix D is dedicated to the proof of Theorem 5.1. The proof reveals strong ties with polyharmonic operators on the unit ball and higher order differential operators on the interval [0, 1]. We refer to [1] for structurally related spectral decompositions of polyharmonic operators on [0, 1] with homogeneous Neumann boundary conditions.

### Corollary 5.3

($d=3$, $p=1$) The nonzero eigenvalues of $T^{3,1}_m$, for $m\in \mathbb {N}\setminus \{0\}$, are exactly $\lambda =-\omega ^{-4}(4m+2)$, where $\omega$ is a positive solution of the equation

$$\begin{aligned} J_{m-\frac{1}{2}}(\omega ) J_{m-\frac{3}{2}}( \mathrm i \omega ) - \mathrm i J_{m-\frac{1}{2}}( \mathrm i \omega ) J_{m-\frac{3}{2}}(\omega )= 0. \end{aligned}$$

The corresponding eigenspaces are 1-dimensional with the representative

5.12 $$\begin{aligned} f_{\lambda }(r)= r^{-\frac{1}{2}}\left( J_{m+\frac{1}{2}}(\omega r)\mathrm i J_{m-\frac{1}{2}}(\mathrm i \omega ) + J_{m+\frac{1}{2}}(\mathrm i \omega r)J_{m-\frac{1}{2}}(\omega )\right) . \end{aligned}$$

The formulas in Corollary 5.3 are derived from Theorem 5.1. Since $J_{m-\frac{1}{2}}(\textrm{i}\omega )\ne 0$, for all $\omega \in \mathbb {R}\setminus \{0\}$, the eigenspaces are 1-dimensional and (5.12) is not the zero-function.

In view of (4.2) in Theorem 4.3 we are particularly interested in kernels of the form $c-\Vert x-y\Vert$. In this case, the expansion holds with $-\mathcal {K}^{d,1}_m$ for $m\ge 1$ and $c-\mathcal {K}^{d,1}_0$ for $m=0$.

## The Rotation Group $\textrm{SO}(3)$

In this section we derive the Fourier expansion of the discrepancy kernel on the special orthogonal group $\textrm{SO}(3)$. The eigenfunctions turn out to be classical functions but the coefficients and their decay rates need to be determined. We also provide numerical experiments by using the nonequispaced fast Fourier transform on $\textrm{SO}(3)$.

### Fourier Expansion on $\textrm{SO}(3)$

By identifying $\mathbb {R}^{d\times d}$ with $\mathbb {R}^{d^2}$, Theorem 4.3 applies to subsets of $\mathbb {R}^{d\times d}$ endowed with the trace inner product

$$\begin{aligned} \langle x,y\rangle _{\textrm{F}}:=\textrm{trace}(x^\top y),\quad x,y\in \mathbb {R}^{d\times d}, \end{aligned}$$

and the induced Frobenius norm $\Vert \cdot \Vert _{\textrm{F}}$ on $\mathbb {R}^{d\times d}$. In this way, $\textrm{SO}(3)$ is contained in $\mathbb {B}^{9}_{\sqrt{3}}$, and it is natural to consider ${s}=\sqrt{3}$. We endow $\textrm{SO}(3)$ with the normalized Haar measure $\sigma _{\textrm{SO}(3)}$. Let $\{\mathcal {D}^m_{k,l}:k,l=-m,\ldots ,m\}$ denote the orthonormal Wigner $\mathcal {D}$-functions on $\textrm{SO}(3)$, which are closely related to the irreducible representations of $\textrm{SO}(3)$ and provide an orthonormal basis for $L_2(\textrm{SO}(3))$, cf. [48]. For $p>0$, the Fourier expansion

6.1 $$\begin{aligned} 2^{-\frac{p}{2}}\Vert x-y\Vert ^p_{\textrm{F}} =\sum _{m=0}^\infty a_m(p,\textrm{SO}(3)) \sum _{k,l=-m}^m \mathcal {D}^m_{k,l}(x) \overline{\mathcal {D}^m_{k,l}(y)},\quad x,y\in \textrm{SO}(3),\nonumber \\ \end{aligned}$$

holds and, analogous to (3.5), the coefficients are

6.2 $$\begin{aligned} a_m(p,\textrm{SO}(3))\!:=\frac{1}{(2m+1)^2}\!\!\!\!\!\iint \limits _{\textrm{SO}(3)\times \textrm{SO}(3)} \!\!\!\!\!\!\!2^{-\frac{p}{2}}\Vert x-y\Vert _{\textrm{F}}^p\!\!\! \sum _{k,l=-m}^m\!\!\! \overline{\mathcal {D}^m_{k,l}(x)}\mathcal {D}^m_{k,l}(y)\textrm{d}\sigma _{\textrm{SO}(3)}(x)\textrm{d}\sigma _{\textrm{SO}(3)}(y).\nonumber \\ \end{aligned}$$

We now compute these coefficients for the entire range $p>-3$.

#### Proposition 6.1

For $p > -3$, the coefficients (6.2) are

6.3 $$\begin{aligned} a_m(p,\textrm{SO}(3)) = \frac{2^p\Gamma (\frac{p}{2}+\frac{3}{2})}{\sqrt{\pi }\Gamma (-\frac{p}{2})} \cdot \frac{\Gamma (-\frac{p}{2}+m)}{\Gamma (\frac{p}{2}+2+m)}\cdot \frac{1}{(m+\frac{1}{2})}. \end{aligned}$$

In particular, if $p\not \in 2\mathbb {N}$, then

$$\begin{aligned} |a_m(p,\textrm{SO}(3))| = \left| \frac{2^p\Gamma (\frac{p}{2}+\frac{3}{2})}{\sqrt{\pi } \Gamma (-\frac{p}{2})}\right| m^{-(p+3)}(1+o(1)),\qquad m\in \mathbb {N}, \end{aligned}$$

and the series (6.1) terminates if $p\in 2\mathbb {N}$.

The proof is given in Appendix E. For $p\in 2\mathbb {N}$, we again apply the convention $\frac{\Gamma (-\frac{p}{2}+m)}{\Gamma (-\frac{p}{2})}=(-\frac{p}{2})_m$ in (6.3), so that $a_m(p,\textrm{SO}(3))=0$ for all $m>\frac{p}{2}$ if $p\in 2\mathbb {N}$. Provided that $\tau >3/2$, the Sobolev space $\mathbb {H}^\tau (\textrm{SO}(3))$ is the reproducing kernel Hilbert space associated with the reproducing kernel

$$\begin{aligned} (x,y)\mapsto \sum _{m=0}^\infty (1+m(m+1))^{-\tau }\sum _{k,l=-m}^m \mathcal {D}^m_{k,l}(x) \overline{\mathcal {D}^m_{k,l}(y)},\quad x,y\in \textrm{SO}(3). \end{aligned}$$

The choice $p=1$ in Proposition 6.1 implies that the kernel $K_{\beta _{9,s}}|_{\textrm{SO}(3)\times \textrm{SO}(3)}$ reproduces the Sobolev space $\mathscr {H}_{K_{\beta _{9,s}}}(\textrm{SO}(3))=\mathbb {H}^{2}(\textrm{SO}(3))$ with an equivalent norm provided that $s\ge \sqrt{3}$.

For $d\ge 2$, we shall observe that $K_{\beta _{d^2,\sqrt{d}}}|_{\textrm{SO}(d)\times \textrm{SO}(d)}$ reproduces the Sobolev space $\mathbb {H}^{\tau }(\textrm{SO}(d))$, for $\tau =\frac{d(d-1)+2}{4}$, with an equivalent norm. Indeed, Theorem 4.3 and Sect. 3 yield that $K_{\beta _{d^2,\sqrt{d}}}|_{\mathbb {S}^{d^2-1}\times \mathbb {S}^{d^2-1}}$ reproduces $\mathbb {H}^{\frac{d^2}{2}}(\mathbb {S}^{d^2-1})$ with equivalent norms. Rescaling implies that $K_{\beta _{d^2,\sqrt{d}}}|_{\sqrt{d}\,\mathbb {S}^{d^2-1}\times \sqrt{d}\,\mathbb {S}^{d^2-1}}$ reproduces $\mathbb {H}^{\frac{d^2}{2}}({\sqrt{d}\,}\mathbb {S}^{d^2-1})$. Since $\textrm{SO}(d)\subset \sqrt{d}\,\mathbb {S}^{d^2-1}$, results on restricting kernels in [27] lead to $\tau =\frac{d^2}{2}-\frac{1}{2}\left( (d^2-1)- \dim (\textrm{SO}(d))\right)$, where $\dim (\textrm{SO}(d))=\frac{d(d-1)}{2}$.

### Numerical Examples on $\textrm{SO}(3)$

Proposition 6.1 yields the coefficients of the kernel expansion

$$\begin{aligned} K_{\beta _{9,\sqrt{3}}}(x,y) = \sum _{m=0}^\infty a_m \sum _{k,l=-m}^m \mathcal {D}^m_{k,l}(x)\overline{\mathcal {D}^m_{k,l}(y)},\quad x,y\in \textrm{SO}(3). \end{aligned}$$

For $\text {supp}(\mu ),\text {supp}(\nu _n)\subset \textrm{SO}(3)$, the $L_2$-discrepancy (1.6) for $K_{\beta _{9,\sqrt{3}}}$ becomes

6.4 $$\begin{aligned} \mathscr {D}_{\beta _{9,\sqrt{3}}}(\mu ,\nu _n) = \sum _{m=0}^\infty a_m \sum _{k,l=-m}^m \left| \hat{\mu }^m_{k,l}- \frac{1}{n}\sum _{j=1}^n \overline{\mathcal {D}^m_{k,l}(x_j)}\right| ^2, \end{aligned}$$

where $\hat{\mu }^m_{k,l}$ denotes the Fourier coefficient of $\mu$ with respect to $\mathcal {D}^m_{k,l}$, cf. (1.6). We truncate the series (6.4) at $M=8$ and minimize

**Fig. 2:**
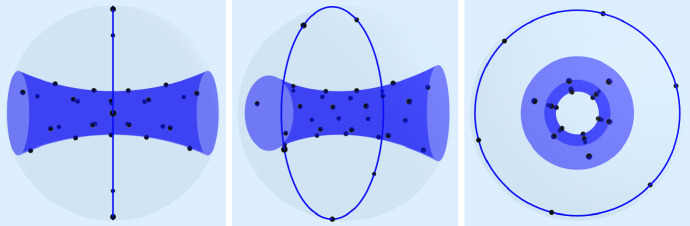
We use the standard parametrization of $\textrm{SO}(3)$ by $\mathbb {S}^3$ via unit quaternions, which is then mapped into $\mathbb {R}^3$ by stereographic projection and $\mathbb {R}^3$ is parametrized by $\mathbb {B}^3$ via $x\mapsto \frac{x}{\Vert x\Vert }\tan (\frac{\pi }{2}\Vert x\Vert )$. The target measure $\mu$ is supported on two disjoint parts with weight ratio 9/1 colored in darker blue by the cylindrical surface and the great circle. Numerical minimization of (6.5) splits 30 points in $\textrm{SO}(3)$ into 27 points on the inner surface and 3 points on the great circle. We plotted 6 points on the great circle but antipodal points correspond to the same point in $\textrm{SO}(3)$

6.5 $$\begin{aligned} \sum _{m=0}^M a_m \sum _{k,l=-m}^m \left| \hat{\mu }^m_{k,l} - \frac{1}{n}\sum _{j=1}^n \overline{\mathcal {D}^m_{k,l}(x_j)}\right| ^2 \end{aligned}$$

among all *n*-point sets $\{x_1,\ldots ,x_n\}\subset \textrm{SO}(3)$ for fixed $n=30$. We efficiently solve the least squares minimization by using the nonequispaced fast Fourier transform on $\textrm{SO}(3)$, cf. [32, 44]. Figure 2 shows the minimizing points mapped onto $\mathbb {B}^3$.

## The Grassmannian $\mathcal {G}_{2,4}$

First, the Fourier expansion of the discrepancy kernel on $\mathcal {G}_{2,4}$ is computed. To prepare for developing the nonequispaced fast Fourier transform on $\mathcal {G}_{2,4}$, we then explicitly parametrize the Grassmannian $\mathcal {G}_{2,4}$ by its double covering $\mathbb {S}^2\times \mathbb {S}^2$. Next, we derive the nonequispaced fast Fourier transform on $\mathbb {S}^2\times \mathbb {S}^2$ and provide numerical minimization experiments on $\mathcal {G}_{2,4}$.

### Fourier Expansion on $\mathcal {G}_{2,4}$

Theorem 4.3 also applies to the Grassmannian

$$\begin{aligned} \mathcal {G}_{2,4}:=\{x\in \mathbb {R}^{4\times 4} : x^\top = x,\; x^2=x,\; {\text {rank}}(x)=2\} \end{aligned}$$

with $s=\sqrt{2}$ when $\mathbb {R}^{4\times 4}$ is identified with $\mathbb {R}^{16}$. To derive the Fourier expansion on $\mathcal {G}_{2,4}$, we require some preparations. We shall use integer partitions $\lambda =(\lambda _1,\lambda _2)\in \mathbb {N}^2$ with $\lambda _1\ge \lambda _2\ge 0$. We also denote $|\lambda |:=\lambda _1+\lambda _2$. The orthogonal group $\textrm{O}(4)$ acts transitively on $\mathcal {G}_{2,4}$ by conjugation and induces the irreducible decomposition

7.1 $$\begin{aligned} L_{2}(\mathcal {G}_{2,4},\sigma _{\mathcal {G}_{2,4}}) = \bigoplus _{\lambda _1\ge \lambda _2\ge 0} H_{\lambda }(\mathcal {G}_{2,4}),\qquad H_{\lambda }(\mathcal {G}_{2,4}) \perp H_{\lambda '}(\mathcal {G}_{2,4}), \quad \lambda \ne \lambda ', \end{aligned}$$

where $\sigma _{\mathcal {G}_{2,4}}$ is the normalized orthogonally invariant measure on $\mathcal {G}_{2,4}$ and $H_{\lambda }(\mathcal {G}_{2,4})$ is equivalent to the irreducible representation ${\mathcal {H}}_{2\lambda }^{4}$ of $\textrm{O}(4)$ with type $2\lambda$, cf. [7, 35]. The normalized eigenfunctions of the Laplace–Beltrami operator on $\mathcal {G}_{2,4}$ form an orthonormal basis for $L_{2}(\mathcal {G}_{2,4})$, and each $H_\lambda (\mathcal {G}_{2,4})$ is contained in the eigenspace $E_{\alpha _\lambda }$ associated with the eigenvalue $\alpha _\lambda = 4(\lambda _1^2+\lambda _2^2+\lambda _1)$, cf. [6–8, 23, 35, 45].

Let $Q_\lambda$ be the reproducing kernel of $H_\lambda (\mathcal {G}_{2,4})$. Any orthonormal basis $\{\varphi _{\lambda ,l}\}_{l=1}^{\dim (\mathcal {H}^{4}_{2\lambda })}$ for $H_{\lambda }(\mathcal {G}_{2,4})$ yields the spectral decomposition

7.2 $$\begin{aligned} Q_\lambda (x,y) = \sum _{l=1}^{\dim (\mathcal {H}^{4}_{2\lambda })}\varphi _{\lambda ,l}(x) \overline{\varphi _{\lambda ,l}(y)},\qquad x,y\in \mathcal {G}_{2,4},\quad \lambda _1\ge \lambda _2\ge 0. \end{aligned}$$

The orthogonal decomposition (7.1) leads to the Fourier expansion

7.3 $$\begin{aligned} 2^{-\frac{p}{2}}\Vert x -y\Vert _{\textrm{F}}^{p}=\sum _{\lambda _1\ge \lambda _2\ge 0}a_\lambda (p,\mathcal {G}_{2,4}) Q_\lambda (x,y),\qquad x,y \in \mathcal {G}_{2,4},\quad p>0. \end{aligned}$$

The coefficients $a_\lambda (p,\mathcal {G}_{2,4})$ in (7.3) are

7.4 $$\begin{aligned} a_\lambda (p,\mathcal {G}_{2,4}) := \frac{1}{\dim (\mathcal {H}^{4}_{2\lambda })}\iint \limits _{\mathcal {G}_{2,4} \times \mathcal {G}_{2,4}}2^{-\frac{p}{2}}\Vert x -y\Vert _{\textrm{F}}^{p} \overline{Q_\lambda (x,y)} \textrm{d}\sigma _{\mathcal {G}_{2,4}}(x)\textrm{d}\sigma _{\mathcal {G}_{2,4}}(y).\nonumber \\ \end{aligned}$$

In order to determine $a_\lambda (p,\mathcal {G}_{2,4})$, we shall make use of the hypergeometric coefficients $(f)_{(\lambda _1,\lambda _2)} := (f)_{\lambda _1} (f-\tfrac{1}{2})_{\lambda _2}$. Also recall the notation $|\lambda |=\lambda _1+\lambda _2$ and $\Vert \lambda \Vert =\sqrt{\lambda _1^2+\lambda _2^2}$.

#### Theorem 7.1

For $p>-4$, we have7.5In particular, if $p\not \in 2\mathbb {N}$, then

7.6 $$\begin{aligned} |a_\lambda (p,\mathcal {G}_{2,4})|= \left| \frac{\Gamma (\frac{p}{2}+2)}{2\Gamma (-\frac{p}{2})}\right| \Vert \lambda \Vert ^{-(p+4)} (1+o(1)), \qquad \lambda _1\ge \lambda _2\ge 0, \end{aligned}$$

and the series (7.3) terminates if $p\in 2\mathbb {N}$.

The proof of this theorem is contained in Section F.1 of Appendix F. If $p\in 2\mathbb {N}$, then $a_\lambda (p,\mathcal {G}_{2,4})=0$ for all $|\lambda |>\frac{p}{2}$. For $\tau >2$, the Sobolev space $\mathbb {H}^\tau (\mathcal {G}_{2,4})$ is the reproducing kernel Hilbert space with associated reproducing kernel

7.7 $$\begin{aligned} (x,y)\mapsto \sum _{\lambda _1\ge \lambda _2\ge 0}(1+4\lambda _1^2+4\lambda _2^2+4\lambda _1)^{-\tau }Q_\lambda (x,y),\quad x,y\in \mathcal {G}_{2,4}, \end{aligned}$$

cf. [11, 16]. Since the coefficients in (7.7) behave asymptotically as $\Vert \lambda \Vert ^{-2\tau }$, the choice $p=1$ in Theorem 7.1 implies that the kernel $K_{\beta _{16,s}}|_{\mathcal {G}_{2,4}\times \mathcal {G}_{2,4}}$ reproduces the Sobolev space $\mathscr {H}_{K_{\beta _{16,s}}}(\mathcal {G}_{2,4})=\mathbb {H}^{\frac{5}{2}}(\mathcal {G}_{2,4})$ with an equivalent norm provided that $s\ge \sqrt{2}$.

Analogous to $\textrm{SO}(d)$ at the end of Sect. 6.1, we deduce with [27] that, for $d\ge 2$, $K_{\beta _{d^2,\sqrt{k}}}|_{\mathcal {G}_{k,d}\times \mathcal {G}_{k,d}}$ reproduces the Sobolev space $\mathbb {H}^{\frac{k(d-k)+1}{2}}(\mathcal {G}_{k,d})$ with an equivalent norm.

### Parametrization of $\mathcal {G}_{2,4}$ by 

To derive the nonequispaced fast Fourier transform on $\mathcal {G}_{2,4}$, we shall first explicitly construct the parametrization of $\mathcal {G}_{2,4}$ by its double covering $\mathbb {S}^2\times \mathbb {S}^2$. We denote the $d\times d$-identity matrix by $I_d$, and the cross-product between two vectors $x,y\in \mathbb {S}^2$ is denoted by $x\times y\in \mathbb {R}^3$. The mapping $\mathcal {P}:\mathbb {S}^2\times \mathbb {S}^2\rightarrow \mathcal {G}_{2,4}$ given by

7.8 $$\begin{aligned} \begin{aligned} (x,y) \mapsto&\frac{1}{2} \begin{pmatrix} 1 + x^\top y &{}&{} - (x \times y)^\top \\ - x \times y &{} &{}x y^\top + y x^\top + (1-x^\top y) I_3 \\ \end{pmatrix} \end{aligned} \end{aligned}$$

is surjective and, for all $x,y,u,v\in \mathbb {S}^2$,

7.9 $$\begin{aligned} \mathcal {P}(u,v)=\mathcal {P}(x,y)\quad \text { if and only if } \quad (u,v) \in \{\pm (x,y) \}, \end{aligned}$$

see Section F.2 and Theorem F.4 of Appendix F. In order to specify the inverse map, note that  can be identified with $\mathcal {M}(3):=\{xy^\top \in \mathbb {R}^{3\times 3} : x,y\in \mathbb {S}^2\}.$ We now define $\mathcal {L}:\mathcal {G}_{2,4}\rightarrow \mathcal {M}(3)$,

7.10 $$\begin{aligned} \mathcal {L}(P) := \left( \begin{array}{ccc} \frac{1}{2}(P_{11}+P_{22}-P_{33}-P_{44}) &{} P_{23} - P_{14} &{} P_{24} + P_{13} \\ P_{23} + P_{14} &{} \frac{1}{2}(P_{11}-P_{22}+P_{33}-P_{44}) &{} P_{34}- P_{12} \\ P_{24} - P_{13} &{} P_{34} + P_{12} &{} \frac{1}{2}(P_{11}-P_{22}-P_{33}+P_{44}) \\ \end{array}\right) ,\nonumber \\ \end{aligned}$$

and direct computations lead to

7.11 $$\begin{aligned} \mathcal {L}(\mathcal {P}(x,y)) = xy^\top ,\quad x,y\in \mathbb {S}^2. \end{aligned}$$

The right-hand side determines *x* and *y* up to the ambiguity (7.9). Under the Frobenius norm, $\mathcal {P}$ is distance preserving in the sense

7.12 $$\begin{aligned} \Vert \mathcal {P}(x,y)-\mathcal {P}(u,v)\Vert _{\textrm{F}} = \Vert xy^\top - uv^\top \Vert _{\textrm{F}},\quad x,y,u,v\in \mathbb {S}^2. \end{aligned}$$

The latter follows from (F.20) in Lemma F.6 in Section F.2 of Appendix F.

We shall now check how the spherical harmonics $Y^m_l$ on $\mathbb {S}^2$ relate to the eigenfunctions $\varphi _{\lambda ,l}\in H_\lambda (\mathcal {G}_{2,4})$ of the Laplace–Beltrami operator on $\mathcal {G}_{2,4}$, cf. (7.2). The functions $Y_{k,l}^{m,n}:\mathcal {G}_{2,4}\rightarrow {\mathbb {C}}$ given by

7.13 $$\begin{aligned} Y_{k,l}^{m,n}(\mathcal {P}(x,y)) := Y_k^m(x) \cdot Y_l^n(y) \end{aligned}$$

are well-defined for $m+n \in 2{\mathbb {N}}$, the latter taking into account the ambiguity (7.9).

#### Theorem 7.2

For $m_\lambda :=(\lambda _1+\lambda _2)$ and $n_\lambda :=(\lambda _1-\lambda _2)$, we have

7.14 $$\begin{aligned} H_{\lambda }(\mathcal {G}_{2,4}) = \textrm{span}\{ Y_{k,l}^{m_\lambda ,n_\lambda }, Y_{l,k}^{n_\lambda ,m_\lambda } : k=-m_\lambda ,\dots ,m_\lambda ,\; l=-n_\lambda ,\dots ,n_\lambda \}.\nonumber \\ \end{aligned}$$

The proof is presented at the end of Section F.2 of Appendix F. Note that the geodesic distance on $\mathcal {G}_{2,4}$ is $\textrm{dist}_{\mathcal {G}_{2,4}}(P,Q)=\sqrt{2}\sqrt{\theta _1^2+\theta _2^2}$, where $\theta _1,\theta _2\in [0,\pi /2]$ are the principal angles determined by the two largest eigenvalues $\cos ^2(\theta _1)$ and $\cos ^2(\theta _2)$ of the matrix *PQ*. Aside from (7.12), $\mathcal {P}$ is also distance-preserving with respect to the respective geodesic distances, i.e.,^4^This equality follows from (F.23) in Lemma F.6 in the appendix via further direct calculations.

The identity (7.14) provides explicit expressions for the orthonormal basis $\{\varphi _{\lambda ,l}\}_{l=1}^{\dim (\mathcal {H}^{4}_{2\lambda })}$ of $H_{\lambda }(\mathcal {G}_{2,4})$ that is used to construct the reproducing kernel $Q_\lambda$ in (7.2). It also provides a fast Fourier transform on $\mathcal {G}_{2,4}$ from the respective transform on $\mathbb {S}^2\times \mathbb {S}^2$ that is developed in the subsequent section.

### Nonequispaced Fast Fourier Transform on $\mathcal {G}_{2,4}$

The nonequispaced fast (spherical) Fourier transform on $\mathbb {S}^2$ has been developed in [39, 41] under the acronym nfsft. Here, we shall derive the analogous transform on $\mathbb {S}^2\times \mathbb {S}^2$, which induces the nonequispaced fast Fourier transform on $\mathcal {G}_{2,4}$ via the mapping $\mathcal {P}$ and (7.14) with (7.13).

For a given finite set of coefficients $f^{m_1,m_2}_{k,l}\in \mathbb {C}$, $m_1,m_2=0,\ldots ,M$, $k=-m_1,\ldots ,m_1$, $l=-m_2,\ldots ,m_2$, we aim to evaluate

7.15 $$\begin{aligned} F(x,y):=\sum _{m_1,m_2=0}^M \sum _{k=-m_1}^{m_1} \sum _{l=-m_2}^{m_2} f^{m_1,m_2}_{k,l} Y^{m_1}_k(x)Y^{m_2}_l(y) \end{aligned}$$

at *n* scattered locations $(x_j,y_j)_{j=1}^n\subset \mathbb {S}^2\times \mathbb {S}^2$. Direct evaluation of (7.15) leads to $O(n M^4)$ operations. We shall now derive an approximative algorithm that is more efficient for $n\gg M$.

By following the ideas in [39, 41], switching to spherical coordinates reveals that (7.15) is a 4-dimensional trigonometric polynomial. This enables the use of the 4-dimensional nonequispaced fast Fourier transform nfft to significantly reduce the complexity. In spherical coordinates the spherical harmonics are trigonometric polynomials such that

7.16 $$\begin{aligned} Y^m_k(z(\theta ,\varphi )) =e^{\textrm{i}k\varphi } \sum _{k'=-m}^m c^m_{k,k'}e^{\textrm{i}k'\theta }, \qquad z(\theta ,\varphi ) = \left( \begin{array}{c} \sin (\theta )\cos (\varphi )\\ \sin (\theta )\sin (\varphi )\\ \cos (\theta ) \end{array} \right) \in \mathbb {S}^2,\nonumber \\ \end{aligned}$$

where $0\le \theta \le \pi$, $0\le \varphi \le 2\pi$, and $c^m_{k,k'}\in \mathbb {C}$ are suitable coefficients that we assume to be given or precomputed. Thus, for $x = z(\theta _1,\varphi _1)$ and $y=z(\theta _2,\varphi _2)$, there are coefficients $b^{k,l}_{k',l'}\in \mathbb {C}$ such that

7.17 $$\begin{aligned} F(x,y)=\sum _{k,l,k',l'=-M}^M b^{k,l}_{k',l'} e^{\textrm{i}k\varphi _1}e^{\textrm{i}l\varphi _2} e^{\textrm{i} k'\theta _1}e^{\textrm{i} l'\theta _2}. \end{aligned}$$

We check in Sect. 1 of Appendix F that the set of coefficients $b^{k,l}_{k',l'}$ can be computed by $O(M^5)$ operations provided that the numbers $c^m_{k,k'}$ in (7.16) are given. The expression (7.17) can be evaluated at *n* scattered locations by the nonequispaced discrete Fourier transform ndft with $O(n M^4)$ operations, cf. [39, 41]. An efficient approximative algorithm is the nonequispaced fast Fourier transform nfft that requires only $O(M^4\log (M)+n|\log (\epsilon )|^4)$ operations with accuracy $\epsilon$, see [39, 41] for details on accuracy. Thus, our algorithm for evaluating (7.15) at *n* scattered locations requires $O(M^5+n|\log (\epsilon )|^4)$ operations. For $n\gg M$, this is a significant reduction in complexity compared to the original $O(nM^4)$ operations. We shall choose $n\sim M^4$ in the subsequent section, so that the complexity is reduced from $O(M^8)$ to $O(M^5+M^4|\log (\epsilon )|^4)$ operations. For potential further reduction, we refer to Remark F.7 in the appendix.

### Numerical example on $\mathcal {G}_{2,4}$

By Theorem 7.1, we can calculate the coefficients of the kernel expansion

$$\begin{aligned} K_{\beta _{16,\sqrt{2}}}(x,y) = \sum _{\lambda _1\ge \lambda _2\ge 0} a_\lambda \sum _{l=1}^{\dim (\mathcal {H}^{d}_{2\lambda })} \varphi _{\lambda ,l}(x)\overline{\varphi _{\lambda ,l}(y)},\quad x,y\in \mathcal {G}_{2,4}. \end{aligned}$$

The eigenfunctions $\varphi _{\lambda ,l}$ are given by the tensor products of spherical harmonics in (7.13), cf. Theorem 7.2. For $\text {supp}(\mu ),\text {supp}(\nu _n)\subset \mathcal {G}_{2,4}$, the $L_2$-discrepancy (1.6) of the kernel $K_{\beta _{16,\sqrt{2}}}|_{\mathcal {G}_{2,4}\times \mathcal {G}_{2,4}}$ is

7.18 $$\begin{aligned} \mathscr {D}_{\beta _{16,\sqrt{2}}}(\mu ,\nu _n) = \sum _{\lambda _1\ge \lambda _2\ge 0} a_\lambda \sum _{l=1}^{\dim (\mathcal {H}^{d}_{2\lambda })} \left| \hat{\mu }_{\lambda ,l} - \frac{1}{n}\sum _{j=1}^n \overline{\varphi _{\lambda ,l}(x_j)}\right| ^2, \end{aligned}$$

where $\hat{\mu }_{\lambda ,l}$ is the Fourier coefficient of $\mu$ with respect to $\varphi _{\lambda ,l}$, cf. (1.6).

Let us consider $\mu =\sigma _{\mathcal {G}_{2,4}}$. According to [11] (see also [15, 16]), the lower bound

$$\begin{aligned} n^{-5/4}\lesssim \mathscr {D}_{\beta _{16,\sqrt{2}}}(\mu ,\nu _n) \end{aligned}$$

holds for all *n*-point sets $\{x_1,\ldots ,x_n\}\subset \mathcal {G}_{2,4}$. We truncate the series (7.18) and let $\nu ^{M}_n=\frac{1}{n}\sum _{j=1}^n\delta _{x^M_j}$ denote a minimizer of

7.19 $$\begin{aligned} \sum _{\lambda +\lambda _2\le M} a_\lambda \sum _{l=1}^{\dim (\mathcal {H}^{d}_{2\lambda })} \left| \hat{\mu }_{\lambda ,l} - \frac{1}{n}\sum _{j=1}^n \overline{\varphi _{\lambda ,l}(x_j)}\right| ^2 \end{aligned}$$

among all *n*-point sets $\{x_1,\ldots ,x_n\}\subset \mathcal {G}_{2,4}$. A suitable choice $n\sim M^4$ leads to the optimal rate

**Fig. 3:**
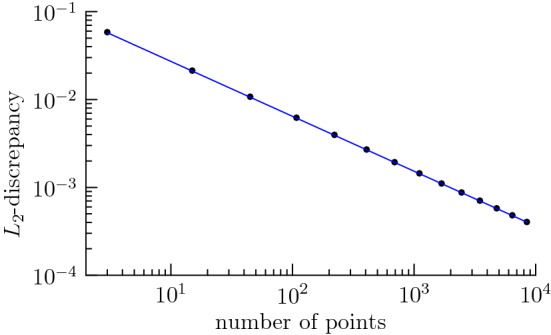
Logarithmic plot of the number of points *n* versus the $L_2$-discrepancy $\mathscr {D}_{\beta _{16},\sqrt{2}}(\mu ,\nu ^{M}_n)$ on $\mathcal {G}_{2,4}$, where $\nu ^{M}_n$ is derived from numerical minimization

7.20 $$\begin{aligned} \mathscr {D}_{\beta _{16,\sqrt{2}}}(\mu ,\nu ^{M}_n)\sim n^{-5/4}, \end{aligned}$$

cf. [11, 25]. Note that we can efficiently solve the least squares minimization (7.19) by using the nonequispaced fast Fourier transform on $\mathcal {G}_{2,4}$ derived from the nonequispaced fast Fourier transform on $\mathbb {S}^2\times \mathbb {S}^2$ of Sect. 7.3 and applying Theorem  7.2. Figure 3 shows logarithmic plots of the number of points versus the $L_2$-discrepancy. We observe a line with slope $-5/4$ as predicted by (7.20).

## Appendix A: Proofs for Section 2

### Proof of Theorem 2.1

Let $h_d:[0,\infty )\rightarrow \mathbb {R}$ denote Euclid’s hat function given by

$$\begin{aligned} h_d(\Vert x\Vert )=\frac{1}{\textrm{vol}(\mathbb {B}^d_{1/2})}(1_{\mathbb {B}^d_{1/2}} * 1_{\mathbb {B}^d_{1/2}})(x),\quad x\in \mathbb {R}^d, \end{aligned}$$

where $1_{\mathbb {B}^d_{1/2}}$ is the indicator function of $\mathbb {B}^d_{1/2}$. For $x,y\in \mathbb {R}^d$ and $t=\Vert x-y\Vert \le 1$, we derive

$$\begin{aligned} K_{d}(x,y)&= \int _0^\infty \frac{1}{\textrm{vol}(\mathbb {B}^d_{r/2})} \int _{\mathbb {R}^d} 1_{\mathbb {B}^d_{r/2}(z)} (x)1_{\mathbb {B}^d_{r/2}(z)}(y) \textrm{d} z \,\textrm{d}G_d(r) =\int _t^{\infty } h_d(t/r)\textrm{d}G_d(r)\\&= \int _t^{1} h_d(t/r)G_d'(r) \textrm{d}r -h_d(t)G_d(1) =t\int _t^{1} r^{-2}h_d'(t/r)G_d(r) \textrm{d}r, \end{aligned}$$

where the last equality is due to partial integration. An explicit expression for $h'_d$ is stated in [29, Equation (11)], so that we obtain

We now compare coefficients of powers of *t* with those of the polynomial $(1-t)^{\frac{d+1}{2}}$. In order to check the coefficient of $t^{2l}$, we first observe

where the last equality makes use of Gauss’ Theorem for the hypergeometric series evaluated at 1 (cf. [47, Equation (1.7.6); Appendix (III.3)]). Direct computation yields

$$\begin{aligned} \frac{(-\frac{d+1}{4})_l (-\frac{d-1}{4})_l}{(-\frac{d}{2})_l} =\left( {\begin{array}{c}\frac{d+1}{2}\\ 2l\end{array}}\right) \frac{(2l)!}{(-4)^{l}} \frac{\Gamma (\frac{d}{2}-l+1)}{\Gamma (\frac{d}{2}+1)}, \end{aligned}$$

so that the coefficient of $$t^{2l}$$ in $$K_{d}(x,y) $$ is

$$\begin{aligned} \left( {\begin{array}{c}\frac{d+1}{2}\\ 2l\end{array}}\right) \frac{(2l)!}{(-4)^l l!} \frac{ \Gamma (\frac{1}{2}-l) }{\Gamma (\frac{d}{2}+1) 2} \frac{d\,\Gamma (\frac{d}{2})}{\sqrt{\pi }} = \left( {\begin{array}{c}\frac{d+1}{2}\\ 2l\end{array}}\right) \frac{(2l)!}{(-4)^{l} l!} \frac{\Gamma (\frac{1}{2}-l) }{ \sqrt{\pi }} =\left( {\begin{array}{c}\frac{d+1}{2}\\ 2l\end{array}}\right) . \end{aligned}$$

Hence, the coefficients for even powers of *t* match. To check the coefficient of $t^{2j+1}$, we first assume $\frac{d-1}{4}\in \mathbb {N}$. The Pfaff–Saalschütz Theorem (cf. [47, Equation (2.3.1.3); Appendix (III.2)]) yields

Thus, the coefficient of $t^{2j+1}$ in $K_{d}(x,y)$ is nonzero if and only if $j\le \frac{d-1}{4}$. Moreover, it is given by

$$\begin{aligned}&\frac{-d\,\Gamma (\frac{d}{2})(-1)^j\left( {\begin{array}{c}\frac{d-1}{2}\\ j\end{array}}\right) (-\frac{d-1}{4})_{\frac{d-1}{4}}(j-\frac{d-1}{2})_{\frac{d-1}{4}} }{\sqrt{\pi }\Gamma (\frac{d+1}{2})(1+2j)(-\frac{d}{2})_{\frac{d-1}{4}}(j-\frac{d-3}{4})_{\frac{d-1}{4}}}\\&\quad =\frac{-\frac{d}{\sqrt{\pi }}\,\Gamma (\frac{d}{2})(-1)^j (\frac{d-1}{4})!(\frac{d+3}{4}-j)_{\frac{d-1}{4}} }{(\frac{d-1}{2}-j)! j!(1+2j)(\frac{d+1}{4}+1)_{\frac{d-1}{4}}(\frac{1}{2}-j)_{\frac{d-1}{4}}}. \end{aligned}$$

Further computations using the duplication formula eventually lead to $$-\left( {\begin{array}{c}\frac{d+1}{2}\\ 2l+1\end{array}}\right) $$. The case $\frac{d+1}{4}\in \mathbb {N}$ is checked analogously. The observation $K_d(x,y)=0$ for $\Vert x-y\Vert >1$ concludes the proof. $\square$

## Appendix B: Proofs for Section 3

### Proof of Proposition 3.2

By expressing the $\mathbb {R}^d$-Fourier transform $\widehat{1_{\mathbb {B}^d_{r}}}(\xi )$ in terms of the Bessel function of the first kind of order *d*/2 and using its asymptotics, we deduce

$$\begin{aligned} \left| \widehat{1_{\mathbb {B}^d_{r}}}(\xi )\right| \lesssim \left( 1+\Vert \xi \Vert ^2\right) ^{-(d+1)/4}, \quad \xi \in \mathbb {R}^d. \end{aligned}$$

Due to the zeros of the Bessel function, the respective lower bound cannot hold. This implies the embedding claims for $\mathbb {H}^{\frac{d+1}{2}}(\mathbb {R}^d)$, in particular, for $d=3$.

To address the restricted kernel, we observe that the value $\textrm{vol}(\mathbb {B}^d_{r/2})K_{d,r}(x,y)$ coincides with the $\mathbb {R}^d$-volume of the two intersecting balls $\mathbb {B}^d_{r/2}(x)\cap \mathbb {B}^d_{r/2}(y)$. This volume has been explicitly computed in [31, Section 2.4.3]. For $d=3$, we obtain

$$\begin{aligned} K_{3,r}(x,y)=1-\frac{3}{2r}\Vert x-y\Vert +\frac{1}{2r^3}\Vert x-y\Vert ^3,\quad x,y\in \mathbb {S}^2, \end{aligned}$$

so that $K_{3,r}|_{\mathbb {S}^{2}\times \mathbb {S}^{2}}$ is a polynomial of degree 3 in $\Vert x-y\Vert$. Its Fourier coefficients $(a_m)_{m\in \mathbb {N}}$ are linear combinations of the Fourier coefficients of the monomial terms, so that (3.7) implies $a_m\lesssim m^{-3}$.

We have checked that there are no cancelations in these linear combinations. Therefore, the asymptotics (3.7) also imply the associated bound from below, which leads to $a_m \sim m^{-3}$. Thus, $K_{3,r}|_{\mathbb {S}^{2}\times \mathbb {S}^{2}}$ reproduces the Sobolev space $\mathbb {H}^{\frac{3}{2}}(\mathbb {S}^{2})$ with equivalent norms. $\square$

## Appendix C: Proofs for Section 4

### Proof of Proposition 4.2

Let us consider $s=1$. The general case follows from rescaling. In order to determine the Fourier expansion of $K_{\beta _{1,1}}|_{[-1,1]\times [-1,1]}$, we need to determine the eigenfunctions with respect to the positive eigenvalues of the integral operator

$$\begin{aligned} T : L_2([-1,1])\rightarrow \mathscr {C}([-1,1]),\quad f\mapsto \int _{-1}^{1} f(t)\left( 1-\tfrac{1}{2}|\cdot -t|\right) \textrm{d}t. \end{aligned}$$

For $\lambda >0$, the equation $T\phi =\lambda \phi$ is equivalent to the associated Sturm–Liouville eigenvalue problem. Indeed, differentiating twice on both sides and carrying out a short calculation, we arrive at the second order homogeneous differential equation

C.1 $$\begin{aligned} \phi ''(x) +\frac{1}{\lambda } \phi (x)=0,\quad x\in (-1,1), \end{aligned}$$

with boundary conditions $\phi '(1) = - \phi '(-1)$ and $\phi (1) + \phi (-1) = -2\phi '(1).$ The general solution of (C.1) is $\phi (x)=c_1\cos (\frac{x}{\sqrt{\lambda }})+c_2\sin (\frac{x}{\sqrt{\lambda }}).$ Direct calculations using the boundary conditions determine $c_1,c_2$ and $\lambda$, so that Mercer’s Theorem and normalization of the eigenfunctions provide the claimed Fourier expansion of the kernel.

Computations analogous to [43, Section 9.5.5] and [22] verify the claimed form of the reproducing kernel Hilbert space. $\square$

## Appendix D: Proofs for Section 5

The two linearly independent eigenfunctions of the differential operator

D.1 $$\begin{aligned} D_m := \partial ^{2}_r + \tfrac{d-1}{r}\partial _r - \tfrac{m(m+d-2)}{r^{2}} \end{aligned}$$

on [0, 1] with respect to a possibly complex eigenvalue $-\omega ^2$ are

$$\begin{aligned} \mathcal {J}^{d,\omega }_{m}(r):=\frac{J_{m+\frac{d}{2}-1}(\omega r )}{r^{\frac{d}{2}-1}}, \quad \text {and }\quad \mathcal {Y}^{d,\omega }_{m}(r):= \frac{Y_{m+\frac{d}{2}-1}(\omega r )}{r^{\frac{d}{2}-1}}, \end{aligned}$$

where $J_\nu$ and $Y_\nu$ are the Bessel functions of first and second type, respectively.

### Lemma D.1

For odd $d\ge 3$, odd $p>1-d$, and $m\in \mathbb {N}$, any eigenfunction of $T_m^{d,p}$ with eigenvalue $\lambda \ne 0$ is a linear combination of $\left\{ \mathcal {J}^{d,\omega _{\ell }}_{m}: \ell =1,\ldots ,\frac{d+p}{2}\right\}$, where

D.2 $$\begin{aligned} \omega _{\ell } = |v|^{\frac{1}{d+p}} {\left\{ \begin{array}{ll} \textrm{e}^{\pi \textrm{i} \frac{2\ell }{d+p}}, &{} (-1)^{\frac{d+p}{2}}v > 0,\\ \textrm{e}^{\pi \textrm{i}\frac{2\ell +1}{d+p}}, &{} (-1)^{\frac{d+p}{2}}v < 0, \end{array}\right. } \end{aligned}$$

and

$$\begin{aligned} v=-\lambda ^{-1} 2^{d+p-2}(d+2m-2)\left( 2-\tfrac{d}{2}\right) _{\frac{d+p}{2}-1} \left( \tfrac{d+p}{2}-1\right) !. \end{aligned}$$

### Proof of Lemma D.1

Up to a constant depending on *d* and *p*, $K^{d,p}$ is the Green’s function of the polyharmonic equation $\Delta ^{\frac{d+p}{2}}u=f$ on $\mathbb {B}^d$ with certain nonlocal boundary conditions, cf. [38]. In particular and by specifying the constant, one deduces that any eigenfunction of $T^{d,p}$ in (5.5) with eigenvalue ${\tilde{\lambda }}\ne 0$ is an eigenfunction of $\Delta ^{\frac{d+p}{2}}$ with eigenvalue

$$\begin{aligned} - {\tilde{\lambda }}^{-1} 2^{d+p-2}\left( 2-\tfrac{d}{2}\right) _{\frac{d+p}{2}-1} \left( \tfrac{d+p}{2}-1\right) ! (d-2)\textrm{vol}(\mathbb {S}^{d-1}). \end{aligned}$$

The Laplacian in polar coordinates is $\Delta = \partial ^2_r + \frac{d-1}{r}\partial _r + \frac{1}{r^2}\Delta _{{\mathbb {S}}^{d-1}}$. The decomposition (5.7) (see also (5.4)) yields $\Delta ^{\frac{d+p}{2}}\varphi ^{d,p}_{m,j,l}=\big (D_{m}^{\frac{d+p}{2}} \varphi ^{d,p}_{m,j}\big )Y^m_l$ on $\mathring{\mathbb {B}}^d$, where $D_m$ is as in (D.1). Since ${\tilde{\lambda }} = \frac{(d-2)\textrm{vol}({\mathbb {S}}^{d-1})}{2m+d-2} \lambda$, any eigenfunction of $T^{d,p}_m$ with eigenvalue $\lambda \ne 0$ is an eigenfunction of $D^{\frac{d+p}{2}}_m$ with eigenvalue

$$\begin{aligned} v=-\lambda ^{-1} 2^{d+p-2}(d+2m-2)\left( 2-\tfrac{d}{2}\right) _{\frac{d+p}{2}-1} \left( \tfrac{d+p}{2}-1\right) !. \end{aligned}$$

The linearly independent eigenfunctions of $D_m^{\frac{d+p}{2}}$ with respect to any eigenvalue $v \ne 0$ are

D.3 $$\begin{aligned} \mathcal {J}^{d,\omega _{\ell }}_{m},\quad \text {and } \quad \mathcal {Y}^{d,\omega _{\ell }}_{m},\qquad \ell =1,\dots ,\tfrac{d+p}{2}, \end{aligned}$$

where

$$\begin{aligned} (-\omega _\ell ^{2})^{\frac{d+p}{2}} = (-1)^{\frac{d+p}{2}} \omega _{\ell }^{d+p} = v, \qquad \ell =1,\dots ,\tfrac{d+p}{2}, \end{aligned}$$

and we take

$$\begin{aligned} \omega _{\ell } := |v|^{\frac{1}{d+p}} {\left\{ \begin{array}{ll} \textrm{e}^{2\pi \textrm{i} \frac{\ell }{d+p}}, &{} (-1)^{\frac{d+p}{2}}v > 0\\ \textrm{e}^{\pi \textrm{i}\frac{2\ell +1}{d+p}}, &{} (-1)^{\frac{d+p}{2}}v < 0, \end{array}\right. } \qquad \ell =1,\dots ,\frac{d+p}{2}. \end{aligned}$$

As an eigenfunction of a positive integer power of the Laplacian, $\phi ^{d,p}_{m,j,l}$ is real analytic on $\mathbb {B}^d$, cf. [5, 36]. Hence, the radial part $\varphi ^{d,p}_{m,j,l}$ must be an analytic function on [0, 1] with even or odd parity for *m* even or odd, respectively, cf. [9]. The functions $\mathcal {Y}^{d,\omega _{\ell }}_{m}$ do not have matching parity, which concludes the proof. $\square$

In order to identify the eigenvalues and the linear combination in Lemma D.1, we check how $T_m^{d,p}$ in (5.8) acts on $\mathcal {J}^{d,\omega }_{m}$.

### Lemma D.2

For *d* and $p>1-d$ odd, we have

D.4

### Proof of Lemma D.2

The idea of the proof is to use the series expansion of the Bessel functions, apply the integral operator to each term, and eventually recover the right-hand side of (D.4). We shall provide the skeleton of the proof and omit some lengthy computations.

Let $d \ge 1$, $p \ge 1-d$ and $r\ge s$. If $d+p$ is even, direct computations yield

$$\begin{aligned} \mathcal {K}_m^{d,p}(r,s) = \frac{(-\frac{p}{2})_{m}}{(\frac{d}{2} - 1)_{m}} \sum _{k=0}^{\frac{d+p}{2}-1} ( -1)^k {\left( {\begin{array}{c}\frac{d+p}{2}-1\\ k\end{array}}\right) } \frac{(-\frac{p}{2}+m)_{k}}{(\frac{d}{2} + m)_{k}} s^{m+2k} r^{p-m-2k}. \end{aligned}$$

We obtain that $( T_m^{d,2l-d} \mathcal {J}^{d,\omega }_{m})(r)$ equals

$$\begin{aligned}&\frac{(\frac{d}{2} -l)_m}{(\frac{d}{2} -1)_m} \!\sum _{k=0}^{l-1} \! \frac{{\left( {\begin{array}{c}l-1\\ k\end{array}}\right) }(\frac{d}{2}-l+m)_{k}}{( -1)^k(\frac{d}{2} + m)_{k}}\\&\quad \Bigg [ \! \frac{\int _0^r J_{m+\frac{d}{2}-1}(\omega s ) s^{\frac{d}{2}+m+2k} \mathrm d s}{r^{2l-d-m-2k}} \!+ \! \frac{\int _r^1 J_{m+\frac{d}{2}-1}(\omega s ) s^{2l-\frac{d}{2}-m-2k}\mathrm ds}{r^{m+2k}} \!\Bigg ]\!. \end{aligned}$$

For $\alpha > 0$, $k \in {\mathbb {N}}_0$, $\omega \in {\mathbb {C}}$ and $r > 0$, integration of each term of the power series of the Bessel function eventually yields

D.5 $$\begin{aligned} \int _0^r J_{\alpha }(\omega s) s^{\alpha + 2k +1} \mathrm d s&= \sum _{i=0}^{k} \frac{2^i (-k)_{i}}{ \omega ^{i+1}} \frac{J_{\alpha + i+1} (\omega r)}{r^{i-\alpha -2k-1}},\end{aligned}$$

D.6 $$\begin{aligned} \int _r^1 J_{\alpha }(\omega s) s^{-\alpha + 2k +1} \mathrm d s&= \sum _{i=0}^{k} \frac{(-2)^{i} (-k)_{i}}{\omega ^{i+1}} \Big (\frac{J_{\alpha -i-1} (\omega r)}{r^{i+\alpha -2k-1}} -J_{\alpha -i-1}(\omega )\Big ), \end{aligned}$$

which follows from direct computations and

for $\alpha \in {\mathbb {R}}$, $k \in {\mathbb {N}}_{0}$, and $n \in {\mathbb {N}}$ with $\alpha \not \in \{-(k+1),-k,\dots ,-n \}$. By applying (D.5) and (D.6), we can express $( T_m^{d,2l-d} \mathcal {J}^{d,\omega }_{m})(r)$ as a sum of Bessel functions of various different orders. Straightforward but lengthy computations combined with the identity

D.7 $$\begin{aligned} J_{\alpha }(x) = \sum _{i,k=0}^{l} \frac{(\frac{x}{2})^{2l+1-i}(-1)^{k}(-k)_{i} }{(\alpha -l)_{l+1}(l-k)! k!} \left[ \frac{(\alpha -l)_{k}J_{\alpha +i+1}(x)}{(\alpha +1)_{k}} +\frac{(\alpha -l)_{l-k} J_{\alpha -i-1}(x)}{(-1)^{l+i}(\alpha +1)_{l-k}} \right] \nonumber \\ \end{aligned}$$

eventually lead to the claimed equality (D.4).

In order to verify (D.7), according to the definition of the Bessel function $J_\alpha (x)$, we have to show that the coefficient of $(x/2)^{\alpha +2m}$ on the right-hand side of (D.7) equals $(-1)^m/(m!\Gamma (m+\alpha +1))$, for $m=0,1,\dots$. Let first $m\ge l$. Then this coefficient $R(\alpha ,m)$ equals

D.8

Both hypergeometric series can be evaluated by means of Gauss’ Theorem. Thus, we obtain

$$\begin{aligned} R(\alpha ,m)=&\sum _{k=0}^{l} \frac{(-1)^{k+m+l+1}}{(\alpha +k-l)_{l+1}(l-k)! k!(m-l-1)!\Gamma (m-l+\alpha )(m-l+k+\alpha )}\\&\quad +\sum _{k=0}^{l} \frac{(-1)^{k+m}}{(\alpha -k)_{l+1} (l-k)! k!(m-l-1)!\Gamma (m-l+\alpha )(m-l+k)}. \end{aligned}$$

Now we reverse the order of summation in the second sum, i.e., we replace *k* by $l-k$ there. Then both sums can be conveniently put together to yield

The hypergeometric function can be evaluated by means of the classical terminating very-well-poised $_5F_4$-summation (cf. [47, Equation (2.3.4.6); Appendix (III.13)]). After some simplification one arrives at the desired expression $(-1)^m/(m!\Gamma (m+\alpha +1))$.

If $m<l$, then the first sum in (D.8) does not contribute anything because of the term $(m-l-1)!$ in the denominator. In the second sum, the summation over *i* may be started at $i=l-m$, which can be evaluated by means of the binomial theorem. The result is zero except if $k=l$. Again, in the end one obtains $(-1)^m/(m!\Gamma (m+\alpha +1))$.

$$\square $$

### Proof of Theorem 5.1

We now combine Lemmas D.2 and D.1. Let the linear combination $f=\sum _{\ell =1}^{\frac{d+p}{2}} c_\ell \mathcal {J}^{d,\omega _{\ell }}_{m}$ be an eigenfunction of $T^{d,p}_m$ with eigenvalue $\lambda$ and let $\omega _{\ell }$ be as in (D.2). We obtain for any $\ell = 1,\dots ,\frac{d+p}{2}$ that

$$\begin{aligned} \begin{aligned} \omega _{\ell }^{d+p}&= (-1)^{\frac{d+p}{2}} v \\&= \lambda ^{-1} 2^{d+p-2}(d+2m-2)(-1)^{\frac{d+p}{2}-1}(2-\tfrac{d}{2})_{\frac{d+p}{2}-1} (\tfrac{d+p}{2}-1)! \\&= \lambda ^{-1} 2^{d+p-2}(d+2m-2)(-\tfrac{p}{2})_{\frac{d+p}{2}-1} (\tfrac{d+p}{2}-1)!, \end{aligned} \end{aligned}$$

and thus

D.9 $$\begin{aligned} T^{d,p}_m f - \lambda f = \frac{(-\frac{p}{2})_m}{(\frac{d}{2} -1)_m} F(r)\!^{\top } A(\omega ) \,c, \end{aligned}$$

where $A(\omega )$ is as in (5.10), $c=(c_{\ell })_{\ell =1,\dots ,\frac{d+p}{2}}$, and

For $i=1,\dots ,\tfrac{d+p}{2}$, the hypergeometric functions in *F* are polynomials of exact degree $d+p-2i$ and thus linearly independent. Hence, for $c \ne 0$, the right-hand side of (D.9) vanishes if and only if $A(\omega )$ is singular and *c* is in its nullspace. $\square$

## Appendix E: Proofs for Section 6

### Proof of Proposition 6.1

We shall derive the coefficients $a_m(p,\textrm{SO}(3))$ from the family of spherical coefficients $a_m(p,\mathbb {S}^1)$. The half-angle identity $\sin (\frac{t}{2})=\sqrt{\frac{1-\cos (t)}{2}}$, for $t\in [0,\pi ]$, implies

E.1 $$\begin{aligned} 2^{-\frac{p}{2}}\Vert x-y\Vert ^p = \sqrt{1-\langle x,y\rangle }^p = 2^{\frac{p}{2}}\sin (\tfrac{s}{2})^p,\qquad x,y\in \mathbb {S}^{d-1}, \end{aligned}$$

where $s=\arccos (\langle x,y\rangle )$. For $d=2$, the addition theorem yields

E.2 $$\begin{aligned} Y^m_1(x)\overline{Y^m_1(y)}+Y^m_2(x)\overline{Y^m_2(y)} =2\mathcal {T}_m(\langle x,y\rangle ),\qquad x,y\in \mathbb {S}^1,\quad m=1,2,\ldots ,\nonumber \\ \end{aligned}$$

where $\mathcal {T}_m$ are the Chebyshev polynomials of the first kind, i.e., $\mathcal {T}_m\big (\cos (s)\big )=\cos (ms)$, for $s\in [0,\pi ]$. The relation (3.4) for $d=2$ with (E.1) and (E.2) leads to

E.3 $$\begin{aligned} 2^{\frac{p}{2}} \sin (\tfrac{s}{2})^p = a_0(p,\mathbb {S}^1) + 2 \sum _{m=1}^\infty a_m(p,\mathbb {S}^1) \mathcal {T}_m\big (\cos (s)\big ),\qquad s\in [0,\pi ],\quad p>0.\nonumber \\ \end{aligned}$$

We now switch to $\textrm{SO}(3)$. For $x,y\in \textrm{SO}(3)$, the relation

$$\begin{aligned} \Vert x-y\Vert _{\textrm{F}}=2^{\frac{1}{2}}\sqrt{3-\textrm{trace}(x^\top y)}, \end{aligned}$$

the choice $s=\arccos (\frac{\textrm{trace}(x^\top y)-1}{2})$, and (E.3) imply

$$\begin{aligned} 2^{-p} \Vert x-y\Vert _{\textrm{F}}^p = 2^{-\frac{p}{2}} \sqrt{3-\textrm{trace}(x^\top y)}^p&= 2^{\frac{p}{2}} \sin (\tfrac{s}{2})^p \\&= a_0(p,\mathbb {S}^1) + 2 \sum _{m=1}^\infty a_m(p,\mathbb {S}^1) \mathcal {T}_m\big (\cos (s)\big )\\&= a_0(p,\mathbb {S}^1) + 2\sum _{m=1}^\infty a_m(p,\mathbb {S}^1) \mathcal {T}_{2m}\big (\cos (\tfrac{s}{2})\big ). \end{aligned}$$

The Legendre polynomials satisfy $\mathcal {T}_m = \frac{1}{2}(\mathcal {C}^1_{m}-\mathcal {C}^1_{m-2})$ with $\mathcal {T}_0 = \mathcal {C}^1_0$ and $\mathcal {C}^0_{-1} = 0$, for $m \in {\mathbb {N}}$, so that we obtain

E.4 $$\begin{aligned} 2^{-p} \Vert x-y\Vert _{\textrm{F}}^p =\sum _{m=0}^\infty (a_{m}(p,\mathbb {S}^1) - a_{m+1}(p,\mathbb {S}^1)) \mathcal {C}^1_{2m}\big (\cos (\tfrac{s}{2})\big ). \end{aligned}$$

We derive (6.3) by calculating the differences $2^{\frac{p}{2}}(a_{m}(p,\mathbb {S}^1) - a_{m+1}(p,\mathbb {S}^1))$ in (E.4) and applying the addition theorem of the orthonormal Wigner $\mathcal {D}$-functions,

E.5 $$\begin{aligned} \sum _{k,l=-m}^m \mathcal {D}_{k,l}^m(x)\overline{\mathcal {D}_{k,l}^m(y)} = (2m+1)\mathcal {C}^1_{2m}\big (\cos (\tfrac{s}{2})\big ), \end{aligned}$$

see [48] for (E.5). Analytic continuation and well-known relations for the gamma function cover the remaining values of $p > -3$.

Standard calculations yield $\frac{\Gamma (m-\frac{p}{2})}{\Gamma (m+\frac{p}{2}+2)}=m^{-2-p}(1+o(1))$, which concludes the proof. $\square$

## Appendix F: Proofs for Section 7

### F.1. Proofs for Section 7.1

#### Proof of Theorem 7.1

Proof of (7.5): According to [20], $Q_\lambda$ is explicitly given in terms of Legendre polynomials by

F.1 $$\begin{aligned} Q_\lambda (x,y) = \frac{c_\lambda }{2} \left( \mathcal {C}^{\frac{1}{2}}_{\lambda _1+\lambda _2}(\xi _{+}) \mathcal {C}^{\frac{1}{2}}_{\lambda _1-\lambda _2}(\xi _{-}) + \mathcal {C}^{\frac{1}{2}}_{\lambda _1+\lambda _2} (\xi _{-})\mathcal {C}^{\frac{1}{2}}_{\lambda _1-\lambda _2}(\xi _{+})\right) , \end{aligned}$$

where $c_\lambda :=\dim (\mathcal {H}^4_{2\lambda })=\left( 2-\delta _{0,\lambda _2}\right) \left( (2\lambda _1+1)^2-4\lambda ^2_2\right)$ and $\theta _1$, $\theta _2$ denote the principal angles between *x* and *y* and

F.2 $$\begin{aligned} \xi _{+} = \cos (\theta _1+\theta _2), \quad \xi _{-} = \cos (\theta _1-\theta _2). \end{aligned}$$

In order to write the integral (7.4) in terms of the variables $\xi _\pm$, we first observe

$$\begin{aligned} 2^{-\frac{p}{2}}\Vert x-y\Vert ^p_{\textrm{F}} = \left( 2- \textrm{trace}(xy)\right) ^{\frac{p}{2}}&= \left( 2-\cos (\theta _1)^2-\cos (\theta _2)^2\right) ^{\frac{p}{2}}\\&= \left( 1-\tfrac{1}{2}\cos (2\theta _1)-\tfrac{1}{2}\cos (2\theta _2)\right) ^{\frac{p}{2}}\\&= \left( 1-\xi _{+}\xi _{-}\right) ^{\frac{p}{2}}. \end{aligned}$$

We set $-q:=p/2$ as well as $m:=\lambda _1+\lambda _2$ and $n:=\lambda _1-\lambda _2$. According to [20], the measure $\mu _{\mathcal {G}_{2,4}}\otimes \mu _{\mathcal {G}_{2,4}}$ in (7.4) turns into $d\xi _{+}d\xi _{-}$ for the variables $\xi _\pm$ on $|\xi _{+}| \le \xi _{-} \le 1$, so that we obtain

$$\begin{aligned} a_\lambda (p,\mathcal {G}_{2,4})&= \int _{0}^{1} \int _{-\xi _{-}}^{\xi _{-}} (1-\xi _{+}\xi _{-})^{-q} \frac{1}{2}\left( \mathcal {C}^{\frac{1}{2}}_n(\xi _{+})\mathcal {C}^{\frac{1}{2}}_m(\xi _{-}) +\mathcal {C}^{\frac{1}{2}}_n(\xi _{-})C^{\frac{1}{2}}_m(\xi _{+})\right) d\xi _{+}d\xi _{-}. \end{aligned}$$

Symmetry arguments yield

$$\begin{aligned} a_\lambda (p,\mathcal {G}_{2,4})&= \frac{1}{4} \int _{-1}^{1} \int _{-1}^{1} (1-xy)^{-q} \frac{1}{2}\left( \mathcal {C}^{\frac{1}{2}}_n(x)\mathcal {C}^{\frac{1}{2}}_m(y) +\mathcal {C}^{\frac{1}{2}}_n(y)\mathcal {C}^{\frac{1}{2}}_m(x)\right) \textrm{d}x \textrm{d} y\\&= \frac{1}{4} \int _{-1}^{1} \int _{-1}^{1} (1-xy)^{-q} \mathcal {C}^{\frac{1}{2}}_n(x)\mathcal {C}^{\frac{1}{2}}_m(y) \textrm{d}x \textrm{d}y. \end{aligned}$$

The series expansion of $(1-xy)^{-q}$ converges absolutely for $q<2$, and the Legendre polynomials $\mathcal {C}^{\frac{1}{2}}_m$ are orthogonal to the monomials $x^k$ for $k<m$ or $m \not \equiv k \!\!\!\mod 2$. Therefore, the orthogonality relations yield

$$\begin{aligned} a_\lambda (p,\mathcal {G}_{2,4})&= \frac{1}{4} \sum _{k=0}^{\infty } \frac{(q)_k}{k!} \int _{-1}^1 \mathcal {C}^{\frac{1}{2}}_m(x)x^k dx \int _{-1}^1 \mathcal {C}^{\frac{1}{2}}_n(y)y^k dy\\&= \frac{1}{4} \sum _{k=0}^{\infty } \frac{(q)_{m+2k}}{(m+2k)!} \int _{-1}^1 \mathcal {C}^{\frac{1}{2}}_m(x)x^{2k+m} dx \int _{-1}^1 \mathcal {C}^{\frac{1}{2}}_n(y)y^{2k+m} dy\\&=\frac{1}{4} \sum _{k=0}^{\infty } \frac{(q)_{m+2k}}{(m+2k)!} c_k^m c_{\tfrac{m-n}{2}+k}^n, \qquad \qquad \text {where } c_k^m := \int _{-1}^1 x^{m+2k} \mathcal {C}^{\frac{1}{2}}_m(x) dx. \end{aligned}$$

The use of the generating function and further calculations lead to

F.3 $$\begin{aligned} c_k^m = \frac{m!\,(m+1)_{2k}}{2^{m-1}(\tfrac{3}{2})_m\,4^k\, k!\, (m+\tfrac{3}{2})_k}. \end{aligned}$$

Application of (F.3) and $(q)_{m+2k}=(q)_m(m+q)_{2k}$ yields

$$\begin{aligned} a_\lambda (p,\mathcal {G}_{2,4})= & {} \frac{1}{4} (q)_m\sum _{k=0}^{\infty } \frac{(m+q)_{2k}}{(m+2k)!} \cdot \frac{m!\,(m+1)_{2k}}{2^{m-1}\,(\tfrac{3}{2})_m\, 4^k\, k!\, (m+\tfrac{3}{2})_k}\\{} & {} \cdot \frac{n!\,(n+1)_{m-n+2k}}{2^{n-1}(\tfrac{3}{2})_n\, 4^{\frac{m-n}{2}+k}\, (\tfrac{m-n}{2}+k)!\, (n+\tfrac{3}{2})_{\tfrac{m-n}{2}+k}}. \end{aligned}$$

By reordering and making use of $n!\,(n+1)_{m-n+2k} = (m+2k)!$, which cancels the identical term in the denominator, we are led to

$$\begin{aligned} a_\lambda (p,\mathcal {G}_{2,4})&= \frac{(q)_m\, m!}{4^{m}\,(\tfrac{3}{2})_m}\sum _{k=0}^{\infty }\frac{1}{ (m+\tfrac{3}{2})_k} \cdot \frac{(m+1)_{2k}}{4^k}\cdot \frac{(m+q)_{2k}}{4^k}\\&\quad \cdot \frac{1}{(\tfrac{m-n}{2}+k)!} \cdot \frac{1}{ (\tfrac{3}{2})_n\,(n+\tfrac{3}{2})_{\tfrac{m-n}{2}+k}} \cdot \frac{1}{k!}\\&= \frac{(q)_m\, m!}{4^{m}\,(\tfrac{3}{2})_m\,(\tfrac{m-n}{2})!\,(\tfrac{3}{2})_n\,(n+\tfrac{3}{2})_{\tfrac{m-n}{2}}}\\&\quad \sum _{k=0}^{\infty }\frac{ (\tfrac{m+1}{2})_{k}\,(\tfrac{m+2}{2})_{k}\,(\tfrac{m+q}{2})_{k}\,(\tfrac{m+q+1}{2})_{k} }{ (m+\tfrac{3}{2})_k\,(\tfrac{m-n+2}{2})_k\,(\tfrac{m+n+3}{2})_k} \cdot \frac{1}{k!}, \end{aligned}$$

where the equality in the last line is due to the identities

$$\begin{aligned} \frac{(m+1)_{2k}}{4^k}&=(\tfrac{m+1}{2})_{k}\,(\tfrac{m+2}{2})_{k},&\frac{(m+q)_{2k}}{ 4^{k}}&= (\tfrac{m+q}{2})_{k}\,(\tfrac{m+q+1}{2})_{k} ,\\ (\tfrac{m-n}{2}+k)!&=(\tfrac{m-n}{2})!\,(\tfrac{m-n+2}{2})_k,&(n+\tfrac{3}{2})_{\tfrac{m-n}{2}+k}&=(\tfrac{m+n+3}{2})_k\,(n+\tfrac{3}{2})_{\tfrac{m-n}{2}}. \end{aligned}$$

The relation $(\tfrac{3}{2})_n (n+\tfrac{3}{2})_{\tfrac{m-n}{2}}=(\tfrac{3}{2})_{\tfrac{m+n}{2}}$ and our choices $m=|\lambda |$, $n=\lambda _1-\lambda _2$ yield $(\tfrac{m-n}{2})!(\tfrac{3}{2})_n (n+\tfrac{3}{2})_{\tfrac{m-n}{2}}=(\tfrac{3}{2})_{\lambda _1}\lambda _2!=(\tfrac{3}{2})_\lambda$, so that $q=-p/2$ and the definition of the ${}_4F_3$ hypergeometric series conclude the proof of (7.5).

Proof of (7.6): The proof of the decay property for $a_\lambda (p,\mathcal {G}_{2,4})$ requires some preparation and auxiliary results. For $p\not \in 2\mathbb {N}$, direct calculations yield

F.4 $$\begin{aligned}{} & {} a_\lambda (p,\mathcal {G}_{2,4}) =\frac{2^{-\frac{p}{2}-3}}{\Gamma \big (-\frac{p}{2}\big ) }\nonumber \\{} & {} \quad \sum _{k=0}^\infty \frac{ \Gamma \big (\frac{|\lambda |}{2}+\frac{1}{2}+k\big )\, \Gamma \big (\frac{|\lambda |}{2}+1+k\big )\, \Gamma \big (\frac{|\lambda |}{2}+\frac{1}{2}-\frac{p}{4}+k\big )\, \Gamma \big (\frac{|\lambda |}{2}-\frac{p}{4}+k\big ) }{\Gamma \big (|\lambda |+\frac{3}{2}+k\big )\, \Gamma \big (\lambda _1+\frac{3}{2}+k\big )\, \Gamma \big (\lambda _2+1+k\big )\, \Gamma \big (k+1\big )}.\nonumber \\ \end{aligned}$$

In the following, we treat the case $\lambda _1=an$ and $\lambda _2= (1-a)n$ for $n\in \mathbb {N}$ and $n\rightarrow \infty$, with an arbitrary $a\in [\frac{1}{2},1]$, so that $|\lambda |=n$. Summarizing the proof of (7.6), we shall first verify that the summand in (F.4),

F.5 $$\begin{aligned} S(n,k):=\frac{ \Gamma \big (\frac{n}{2}+\frac{1}{2}+k\big )\, \Gamma \big (\frac{n}{2}+1+k\big )\, \Gamma \big (\frac{n}{2}+\frac{1}{2}-\frac{p}{4}+k\big )\, \Gamma \big (\frac{n}{2}-\frac{p}{4}+k\big ) }{ \Gamma \big (n+\frac{3}{2}+k\big )\, \Gamma \big (an+\frac{3}{2}+k\big )\, \Gamma \big ((1-a)n+1+k\big )\, \Gamma \big (k+1\big )},\nonumber \\ \end{aligned}$$

as a sequence in *k*, is unimodal, i.e., it first increases until it has reached its maximum and then decreases, see Lemma F.2. Second, approximation of *S*(*n*, *k*) for $k\ge \varepsilon n^2$ with the help of Stirling’s formula, where $\varepsilon >0$ but fixed, leads to an asymptotic formula for $\sum _{k\ge \varepsilon n^2}S(n,k)$; see Lemma F.3. Third, we let $\varepsilon \rightarrow 0$ to obtain the asymptotic behavior of the full sum $\sum _{k\ge 0}S(n,k)$ for $n\rightarrow \infty$; see (F.8). If the result is substituted in (F.4), the claimed decay in (7.6) follows immediately upon observing $\Vert \lambda \Vert ^2=(2a^2-2a+1)n^2$.

To start with, we may consider *S*(*n*, *k*) as a function of real *k*.

#### Lemma F.1

Given $n\in \mathbb {N}$, let $k_0=k_0(n)\in (0,\infty )$ be such that $\frac{\partial }{\partial k}S(n,k_0)=0$. Then

F.6 $$\begin{aligned} k_0=k_0(n)=\frac{2a^2-2a+1}{p+6}n^2+O(n), \quad \text {for }n\rightarrow \infty . \end{aligned}$$

#### Lemma F.2

If *n* is large enough, *S*(*n*, *k*) has a unique — local and global — maximum for $k\in [0,\infty )$.

#### Lemma F.3

Let $\varepsilon >0$ be fixed and let $A:=\frac{2a^2-2a+1}{p+6}$. Then

$$\begin{aligned} \sum _{k\ge \varepsilon n^2}S(n,k)&=A^{-\frac{p+4}{2}} n^{-(p+4)} \big (1+O(\tfrac{1}{n})\big )\\&\quad \int _{-1+\frac{\varepsilon }{A}}^\infty (1+x)^{-\frac{p+6}{2}} \exp \big (-\tfrac{p+6}{2(1+x)}\big )\textrm{d}x, \quad \text {for }n\rightarrow \infty . \end{aligned}$$

We postpone the proofs of Lemmas F.1, F.2, and F.3, and discuss their consequences first. If we let $\varepsilon \rightarrow 0$ and substitute $t=\frac{p+6}{2(1+x)}$ in the above integral, then the integral definition of the gamma function yields

F.7 $$\begin{aligned} \int _{-1}^\infty \left( 1+x\right) ^{-\frac{p+6}{2}} \exp \Big ( -\frac{p+6}{2\left( 1+x\right) }\Big )\textrm{d}x =\Big (\frac{p+6}{2}\Big )^{-\frac{p+6}{2}}\, \Gamma \Big (\frac{p+4}{2}\Big ). \end{aligned}$$

Lemma F.3 and (F.7) provide the asymptotic lower bound on $\sum _{k\ge 0}S(n,k)$ of the following two-sided claim:

F.8 $$\begin{aligned} \sum _{k\ge 0}S(n,k)= \Big (\frac{2}{2a^2-2a+1}\Big )^{\frac{p+4}{2}} \Gamma \Big (\frac{p+4}{2}\Big )n^{-p-4} \left( 1+o(1)\right) , \quad \text {for }n\rightarrow \infty .\nonumber \\ \end{aligned}$$

To verify the asymptotic upper bound on $\sum _{k\ge 0}S(n,k)$ in (F.8), we observe

$$\begin{aligned} \sum _{k\ge 0}S(n,k)\le \sum _{k\ge \varepsilon n^2}S(n,k) +\varepsilon n^2 S(n,\varepsilon n^2) ,\quad \varepsilon <A, \end{aligned}$$

which is due to Lemma F.2, saying that *S*(*n*, *k*) grows until its maximum at $k=k_0\sim An^2$. By Lemma F.3 and letting $\varepsilon \rightarrow 0$, we obtain the upper bound in (F.8). By taking into account the additional factor $2^{-\frac{p}{2}-3}/\Gamma \big (-\frac{p}{2}\big )$ in (F.4) and the relation $\Vert \lambda \Vert ^2=(2a^2-2a+1)n^2$, we observe that (F.8) provides our claim (7.6).

To complete the proof of (7.6) in Theorem 7.1, it remains to prove Lemmas F.1, F.2, and F.3.

#### Proof of Lemma F.1

The condition $0=\frac{\partial }{\partial k}S(n,k_0)$ implies $0=\frac{\partial }{\partial k}\big (\log S(n,k_0)\big )$. By using the digamma function $\psi (z)$, the logarithmic derivative of (F.5) can be written as

$$\begin{aligned} \frac{\partial }{\partial k}\big (\log S(n,k)\big )= & {} \psi \Big (\frac{n}{2}+\frac{1}{2}+k\Big ) +\psi \Big (\frac{n}{2}+1+k\Big ) +\psi \Big (\frac{n}{2}+\frac{1}{2}-\frac{p}{4}+k\Big )\\{} & {} +\psi \Big (\frac{n}{2}-\frac{p}{4}+k\Big ) -\psi \Big (n+\frac{3}{2}+k\Big ) \\{} & {} -\psi \Big (an+\frac{3}{2}+k\Big ) -\psi \big ((1-a)n+1+k\big ) -\psi (k+1). \end{aligned}$$

For $k=o(n)$, the above expression is certainly positive for large *n*, hence nonzero. If the order of magnitude of *k* is at least the one of *n* (in symbols, $n=O(k)$), then we may estimate the logarithmic derivative by

F.9 $$\begin{aligned} \frac{\partial }{\partial k}\big (\log S(n,k)\big )= & {} \log \big (\frac{n}{2}+\frac{1}{2}+k\big ) +\log \big (\frac{n}{2}+1+k\big ) +\log \big (\frac{n}{2}+\frac{1}{2}-\frac{p}{4}+k\big )\nonumber \\{} & {} \quad +\log \big (\frac{n}{2}-\frac{p}{4}+k\big )\nonumber \\{} & {} -\log \big (n+\frac{3}{2}+k\big ) -\log \big (an+\frac{3}{2}+k\big )\nonumber \\{} & {} \quad -\log \big ((1-a)n+1+k\big ) -\log \big (k+1\big ) +O\big (n^{-1}\big ), \end{aligned}$$

where the asymptotics $\psi (x)=\log x-\frac{1}{2x}+O\big (x^{-2}\big )$, for $x\rightarrow \infty$, cf. [24, Equation 1.18(7)]), were used. By applying the exponential function on both sides of $\frac{\partial }{\partial k}\big (\log S(n,k_0)\big )=0$, together with the above estimation for $\frac{\partial }{\partial k}\big (\log S(n,k)\big )$ we obtain

F.10 $$\begin{aligned} Q(n,k_0)=1+O(n^{-1}), \end{aligned}$$

where

$$\begin{aligned} Q(n,k):=\frac{ \big (\frac{n}{2}+\frac{1}{2}+k\big ) \big (\frac{n}{2}+1+k\big ) \big (\frac{n}{2}+\frac{1}{2}-\frac{p}{4}+k\big ) \big (\frac{n}{2}-\frac{p}{4}+k\big )}{ \big (n+\frac{3}{2}+k\big ) \big (an+\frac{3}{2}+k\big ) \big ((1-a)n+1+k\big ) (k+1)}. \end{aligned}$$

For $k\sim cn$, we observe

$$\begin{aligned} \lim _{n\rightarrow \infty }Q(n,k)=\frac{\big (c+\frac{1}{2}\big )^4}{(c+1)(c+a)(c+1-a)c}>1, \end{aligned}$$

so that we may assume that $k_0$ is of larger asymptotic order of magnitude than *n*.

Define *P*(*n*, *k*) by

F.11 $$\begin{aligned} Q(n,k)=1-\frac{P(n,k)}{ \big (n+\frac{3}{2}+k\big ) \big (an+\frac{3}{2}+k\big ) \big ((1-a)n+1+k\big ) (k+1)}. \end{aligned}$$

For *k* of larger asymptotic order of magnitude than *n*, the asymptotics of the digamma function implies that the error term $O(n^{-1})$ in (F.9) and (F.10) may be replaced by $O(k^{-2})$ and hence by $o(k^{-1})$. The relations (F.10) and (F.11) lead to $P(n,k_0)=o(k_0^3)$. Since direct computations yield

F.12 $$\begin{aligned} P(n,k) =\Big (\frac{p+6}{2}\Big ) k^3 - \Big (a^2 -a +\frac{1}{2}\Big )(k^2n^2+k n^3) -\frac{1}{16} n^4 + \text { lower order terms},\nonumber \\ \end{aligned}$$

asymptotically leading terms in *P*(*n*, *k*) must cancel each other. We have already excluded $k_0\sim cn$, so that we now consider $k_0\sim cn^2$ for an appropriate constant *c*. The terms $k^3$ and $k^2n^2$ in *P*(*n*, *k*) must cancel each other, so that

$$\begin{aligned} \frac{1}{2}(p+6)c^3-\frac{1}{2}(2a^2-2a+1)c^2=0. \end{aligned}$$

The solution $c=\frac{1}{p+6}(2a^2-2a+1)$ yields the leading term in (F.6). In order to derive the *O*(*n*) term in (F.6), we have to perform “bootstrap”, i.e., we substitute $k_0=cn^2+k_1$ in (F.12) and apply analogous arguments to eventually conclude $k_1=O(n)$. $\square$

#### Proof of Lemma F.2

We already saw in the previous proof that $\frac{\partial }{\partial k}\log S(n,0)>0$, for sufficiently large *n*. Hence, $\frac{\partial }{\partial k} S(n,0)>0$ holds, so that *S*(*n*, *k*) does not have a local maximum in $k=0$. Convergence of the series (F.4) implies $S(n,k)\rightarrow 0$ for integers $k\rightarrow \infty$. Thus, for sufficiently large *n*, *S*(*n*, *k*) attains a local maximum at some $k_0\in (0,\infty )$. Lemma F.1 implies $k_0\sim \frac{1}{p+6}(2a^2-2a+1)n^2$. In order to investigate *S*(*n*, *k*) in a neighborhood of $k_0$, we compute $\frac{\partial ^2}{\partial s^2}\log S(n,k_0+s)$, which is

F.13 $$\begin{aligned} \begin{aligned} \psi ^{(1)}\big (\frac{n}{2}+\frac{1}{2}+k\big ) +\psi ^{(1)}\big (\frac{n}{2}+1+k\big ) +\psi ^{(1)}\big (\frac{n}{2}+\frac{1}{2}-\frac{p}{4}+k\big ) +\psi ^{(1)}\big (\frac{n}{2}-\frac{p}{4}+k\big )\\ -\psi ^{(1)}\big (n+\frac{3}{2}+k\big ) -\psi ^{(1)}\big (an+\frac{3}{2}+k\big ) -\psi ^{(1)}\big ((1-a)n+1+k\big ) -\psi ^{(1)}\big (k+1\big ), \end{aligned}\nonumber \\ \end{aligned}$$

where $k=k_0+s$ and $\psi ^{(1)}(x)$ denotes the derivative of $\psi (x)$. We claim that, for $\vert s\vert =o( n^2)$ and sufficiently large *n*, we have $\frac{\partial ^2}{\partial s^2}\log S(n,k_0+s)<0$. In order to establish this claim, we make use of $\psi ^{(1)}(x)=\frac{1}{x}+\frac{1}{2x^2}+O(x^{-3})$, for $x\rightarrow \infty$, cf. [24, Equations 1.16(9) and 1.18(9)]), to estimate the individual expressions in (F.13). Using $k=k_0+s=k_0+o(n^2)= k_0+o( k_0)$, we obtain

$$\begin{aligned} \psi ^{(1)}\Big (\frac{n}{2}+\frac{1}{2}+k\Big )&=\frac{1}{\frac{n}{2}+\frac{1}{2}+k} +\frac{1}{2\left( \frac{n}{2}+\frac{1}{2}+k\right) ^2}+O\big (k_0^{-3}\big )\\&=\frac{1}{k\big (1+\frac{\frac{n}{2}+\frac{1}{2}}{k}\big )}+\frac{1}{2 k_0^2}+o\big (k_0^{-2}\big )\\&=\frac{1}{k}\Big (1-\frac{\frac{n}{2}+\frac{1}{2}}{k} +\frac{n^2}{4k^2}\Big ) +\frac{1}{2 k_0^2}+o\big (k_0^{-2}\big ), \end{aligned}$$

where we have used $\frac{1}{1+x}=1-x+x^2+O(x^3)$, for $x\rightarrow 0$. We treat the other terms in (F.13) analogously. Note that $k=k_0+o(n^2)$ also implies $k= \frac{1}{p+6}(2a^2-2a+1)n^2+o(n^2)$, so that, by putting the individual estimates together, we arrive at

$$\begin{aligned} \frac{\partial ^2}{\partial s^2}\log S(n,k_0+s)&= \frac{\frac{p}{2}+3}{k^2} +\frac{n^2}{k^3}\left( -a^2-(1-a)^2\right) +o\big ( k_0^{-2}\big )\\&=-\frac{\frac{p}{2}+3}{k^2}+o\big (k_0^{-2}\big ) =-\frac{\frac{p}{2}+3}{k_0^2}+o\big (k_0^{-2}\big ). \end{aligned}$$

For sufficiently large *n* (and hence large $k_0$), this is evidently negative, as claimed. Thus, *S*(*n*, *k*) has a strict local maximum in $k_0$.

Moreover, say there are $k_0, k'_0\in (0,\infty )$, where *S*(*n*, *k*) has a local maximum, then Lemma F.1 implies that the magnitude of $|k_0- k_0'|$ is of smaller order than $n^2$. The above considerations imply $k_0=k_0'$, which completes the proof. $\square$

To prove Lemma F.3, we shall approximate the summand *S*(*n*, *k*), given in (F.5), with the help of Stirling’s formula (F.14). It turns out that, under this approximation, the sum $\sum _{k\ge 0}S(n,k)$ can then be interpreted as a Riemann integral.

#### Proof of Lemma F.3

Let $k_0$ be the unique location of the maximum of *S*(*n*, *k*). We recall that Lemma F.1 yields $k_0=An^2+O(n)$. We consider $\log S(n,k)$, for $k\ge \varepsilon n^2$, and write $k=k_0+s$, so that $s\ge -k_0+\varepsilon n^2$. Stirling’s formula

F.14 $$\begin{aligned} \log \big (\Gamma (z)\big )=\Big (z-\frac{1}{2}\Big )\log (z)-z+\frac{1}{2}\log (2\pi ) +O\big (z^{-1}\big ) \end{aligned}$$

leads to the estimates

$$\begin{aligned} \log \Big (\Gamma \Big (\frac{n}{2}+\frac{1}{2}+k\Big )\Big ) =&\Big (\frac{n}{2}+k_0+s\Big ) \log \Big (\frac{n}{2}+\frac{1}{2}+k_0+s\Big )\\&\quad -\Big (\frac{n}{2}+\frac{1}{2}+k_0+s\Big )+\frac{1}{2}\log (2\pi ) +O\big (n^{-2}\big )\\ =&\Big (\frac{n}{2}+k_0+s\Big )\left[ \log (k_0+s) + \log \Big (1+\frac{\frac{n}{2}+\frac{1}{2}}{k_0+s}\Big )\right] \\&\quad -\Big (\frac{n}{2}+\frac{1}{2}+k_0+s\Big )+\frac{1}{2}\log (2\pi ) +O\big (n^{-2}\big ), \end{aligned}$$

where we have factored out $k_0+s$. By applying $\log (1+x)=x-\frac{x^2}{2}+O(x^3)$, we obtain

$$\begin{aligned} \log \Big (\Gamma \Big (\frac{n}{2}+\frac{1}{2}+k\Big )\Big )&= \Big (\frac{n}{2}+k_0+s\Big )\\&\quad \left[ \log (k_0) + \log \Big (1+\frac{s}{k_0}\Big ) +\frac{\frac{n}{2}+\frac{1}{2}}{k_0+s} -\frac{1}{2}\Big (\frac{\frac{n}{2}+\frac{1}{2}}{k_0+s}\Big )^2\right] \\&\quad -\Big (\frac{n}{2}+\frac{1}{2}+k_0+s\Big )+\frac{1}{2}\log (2\pi ) +O\big (n^{-1}\big ). \end{aligned}$$

Here, terms such as $\frac{n^3}{(k_0+s)^2}$ or $\frac{n}{k_0+s}$ are of the order $O(n^ {-1})$ and can therefore be subsumed in the error term. Thus, we obtain

$$\begin{aligned} \log \Big (\Gamma \Big (\frac{n}{2}+\frac{1}{2}+k\Big )\Big )=&\Big (\frac{n}{2}+k_0+s\Big )\left[ \log (k_0) + \log \Big (1+\frac{s}{k_0}\Big )\right] +\frac{1}{2}\frac{(\frac{n}{2})^2}{(k_0+s)}\\&\quad -(k_0+s)+\frac{1}{2}\log (2\pi ) +O\big (n^{-1}\big ). \end{aligned}$$

The reasoning for the $O(\,.\,)$-terms are based on our restriction to $k_0+s\ge \varepsilon n^2$. However, the constants in these error terms do contain $\varepsilon$.

For the other gamma functions in (F.5), we proceed similarly. If everything is put together, then we obtain

$$\begin{aligned} \log \big (S(n,k)\big )&=-\frac{p+6}{2}\left[ \log (k_0)+\log \Big (1+\frac{s}{k_0}\Big )\right] -\frac{1}{2}\frac{n^2(2a^2-2a+1)}{(k_0+s)} +O\big (n^{-1}\big )\\&=-\frac{p+6}{2}\left[ \log (k_0)+\log \Big (1+\frac{s}{k_0}\Big )\right] -\frac{p+6}{2\big (1+\frac{s}{k_0}\big )} +O\big (n^{-1}\big ). \end{aligned}$$

For the sum of the *S*(*n*, *k*), we have

F.15 $$\begin{aligned} \sum _{k\ge \varepsilon n^2}S(n,k) =\!\!\!\sum _{s\ge -k_0+\varepsilon n^2} \!\!\!\! k_0^{-\frac{p+6}{2}} \Big (1+\frac{s}{k_0}\Big )^{-\frac{p+6}{2}} \exp \Big ( \frac{-(p+6)}{2\big (1+\tfrac{s}{k_0}\big )}\Big ) \Big (1+O\big (n^{-1}\big )\Big ).\nonumber \\ \end{aligned}$$

By understanding, the sum over *s* is taken over those *s*, for which $k_0+s$ is an integer. The error of the Riemann sum approximation

F.16 $$\begin{aligned} \frac{1}{k_0}\! \sum _{s\ge -k_0+\varepsilon n^2} \Big (1+\frac{s}{k_0}\Big )^{-\frac{p+6}{2}} \exp \Big ( \frac{-(p+6)}{2\big (1+\frac{s}{k_0}\big )}\Big ) \approx \int _{-1+\frac{\varepsilon }{A}}^\infty \!\!\! (1+x)^{-\frac{p+6}{2}} \exp \Big (\frac{-(p+6)}{2(1+x)}\Big )\textrm{d}x\nonumber \\ \end{aligned}$$

is of the order of magnitude $O(k_0^{-1})$. This follows from the fact that the summand in the sum attains a unique local and global maximum — namely at $s=0$ — and therefore the error is bounded above by $k_0^{-1}$ times the absolute variation of the summand — which equals twice the maximum. Substitution of all this in (F.15) concludes the proof. $\square$

Our proof of (7.6) in Theorem 7.1 is now complete. $\square$

### F.2. Proofs for Section 7.2

Recall that the action of $\textrm{SO}(3)\times \textrm{SO}(3)$ on $\mathbb {S}^2\times \mathbb {S}^2$ by left multiplication is transitive, and $\textrm{SO}(4)$ acts transitively on $\mathcal {G}_{2,4}$ by conjugation. For each $O\in \textrm{SO}(4)$, there are $a,b\in \mathbb {S}^3$ such that $O=L_a R_b$, where the left and right isoclinic rotations are

$$\begin{aligned} L_a := \begin{pmatrix} a_1 &{} -a_2 &{} -a_3 &{} -a_4 \\ a_2 &{} a_1 &{} -a_4 &{} a_3 \\ a_3 &{} a_4 &{} a_1 &{} -a_2 \\ a_4 &{} -a_3 &{} a_2 &{} a_1 \\ \end{pmatrix}^\top , \quad R_b := \begin{pmatrix} b_1 &{} -b_2 &{} -b_3 &{} -b_4 \\ b_2 &{} b_1 &{} b_4 &{} -b_3 \\ b_3 &{} -b_4 &{} b_1 &{} b_2 \\ b_4 &{} b_3 &{} -b_2 &{} b_1 \\ \end{pmatrix}. \end{aligned}$$

For $a\in \mathbb {S}^3$, the Euler–Rodrigues formula yields

$$\begin{aligned} S_a:= \begin{pmatrix} a_1^2+a_2^2-a_3^2-a_4^2 &{} 2(a_2a_3-a_1a_4) &{} 2(a_2a_4+a_1a_3) \\ 2( a_2a_3+a_1a_4) &{} a_1^2-a_2^2+a_3^2-a_4^2 &{} 2( a_3a_4-a_1a_2) \\ 2( a_2a_4-a_1a_3) &{} 2(a_3a_4+a_1a_2) &{} a_1^2-a_2^2-a_3^2+a_4^2 \\ \end{pmatrix}^\top \in \textrm{SO}(3). \end{aligned}$$

We are looking for $\mathcal {P}:\mathbb {S}^2\times \mathbb {S}^2\rightarrow \mathcal {G}_{2,4}$ satisfying

F.17 $$\begin{aligned} (L_a R_b)\cdot \mathcal {P}(x,y)\cdot (L_a R_b)^\top =\mathcal {P}(S_a x,S_b y),\qquad a,b\in \mathbb {S}^3,\quad x,y\in \mathbb {S}^2. \end{aligned}$$

#### Theorem F.4

There are exactly two mappings $\mathbb {S}^2\times \mathbb {S}^2\rightarrow \mathcal {G}_{2,4}$ satisfying (F.17). One is $\mathcal {P}$ as in (7.8), and the other is $I_4-\mathcal {P}$. In particular, $\mathcal {P}$ is surjective and, for all $x,y,u,v\in \mathbb {S}^2$,

F.18 $$\begin{aligned} \mathcal {P}(u,v)=\mathcal {P}(x,y)\quad \text {if and only if} \quad (u,v) \in \{\pm (x,y) \}. \end{aligned}$$

#### Proof of Theorem F.4

The identity (F.17) for the specific choice of $\mathcal {P}$ is verified by expanding both sides of the equality and comparing the polynomial expressions. We omit the straightforward but lengthy computation.

Since the conjugate action of $\textrm{SO}(4)$ on $\mathcal {G}_{2,4}$ is transitive, the identity (F.17) also implies surjectivity. Since left and right eigenspaces of $\mathcal {L}(\mathcal {P}(x,y))$ in (7.10) and (7.11) are uniquely determined, we deduce that (F.18) holds.

Let us now address the uniqueness statement. For $a:=(\alpha ,0,0)$, $b:=(\beta ,0,0)$ with $\alpha ,\beta \in \mathbb {S}^1$, we obtain the isoclinic rotations

$$\begin{aligned} L_a = \begin{pmatrix} A_\alpha &{}0\\ 0&{}A_\alpha \end{pmatrix}, \qquad R_b = \begin{pmatrix} B_\beta &{}0\\ 0&{}B^\top _\beta \end{pmatrix}, \end{aligned}$$

where $$A_\alpha =\left( \begin{array}{cc}\alpha _1&{}-\alpha _2\\ \alpha _2&{}\alpha _1\end{array}\right) ,\;B_\beta = \left( \begin{array}{cc} \beta _1&{}-\beta _2\\ \beta _2&{}\beta _1 \end{array}\right) \in \textrm{SO}(2)$$. Since $S_ae_1=S_b e_1=e_1$, any mapping $\tilde{\mathcal {P}}:\mathbb {S}^2\times \mathbb {S}^2\rightarrow \mathcal {G}_{2,4}$ satisfying (F.17) must obey

F.19 $$\begin{aligned} L_a R_b\tilde{\mathcal {P}}(e_1,e_2)(L_a R_b)^\top = \tilde{\mathcal {P}}(e_1,e_2),\quad \alpha ,\beta \in \mathbb {S}^1. \end{aligned}$$

Let *V* denote the range of $\tilde{\mathcal {P}}(e_1,e_2)$, which is a two-dimensional subspace of $\mathbb {R}^4$. The relation (F.19) means that $L_aR_b$ maps *V* into itself, i.e., $L_aR_b V = V$. For all $U_1,U_2\in \textrm{SO}(2)$, there are $\alpha ,\beta \in \mathbb {S}^1$ such that

$$\begin{aligned} L_aR_b = \begin{pmatrix} A_\alpha B_\beta &{}0\\ 0&{}A_\alpha B_\beta ^\top \end{pmatrix} = \begin{pmatrix} U_1&{}0\\ 0&{}U_2 \end{pmatrix}, \end{aligned}$$

so that *V* must either coincide with $\textrm{span}\{e_1,e_2\}$ or with $\textrm{span}\{e_3,e_4\}$. Hence, $\tilde{\mathcal {P}}(e_1,e_2)$ must coincide with either $\textrm{diag}(1,1,0,0)$ or $\textrm{diag}(0,0,1,1)$. It follows from (F.17) and $\textrm{SO}(3)$ acting transitively on $\mathbb {S}^2$ that in the first case $\tilde{\mathcal {P}}=\mathcal {P}$ and in the latter $\tilde{\mathcal {P}}=I_4-\mathcal {P}$.

$$\square $$

#### Remark F.5

The Grassmannian $\mathcal {G}_{2,4}$ has been parametrized in [20] by means of angles but the explicit identity (F.17) that steers our present approach has not been considered there. Our parametrization is compatible with the one used in [19].

The following properties of $\mathcal {P}$ are useful.

#### Lemma F.6

The probability measure $\sigma _{\mathcal {G}_{2,4}}$, induced by the Haar measure on $\textrm{O}(4)$, is the push-forward measure of $\sigma _{{\mathbb {S}}^2}\otimes \sigma _{{\mathbb {S}}^2}$ under $\mathcal {P}$, and we have, for all $x,y,u,v\in \mathbb {S}^2$,

F.20 $$\begin{aligned} \langle \mathcal {P}(x,y),\mathcal {P}(u,v)\rangle _{\textrm{F}}&=1+\langle xy^\top ,uv^\top \rangle _{\textrm{F}}=1 + \langle x,u\rangle \langle y,v\rangle , \end{aligned}$$

F.21 $$\begin{aligned} \mathcal {I}_-\cdot \mathcal {P}(x,y)\cdot \mathcal {I}_-^\top&= \mathcal {P}(y,x), \end{aligned}$$

F.22 $$\begin{aligned} I_4-\mathcal {P}(x,y)&= \mathcal {P}(-x,y) = \mathcal {P}(x,-y), \end{aligned}$$

F.23 $$\begin{aligned} \{\langle x,u\rangle ,\langle y,v\rangle \}&= \pm \{ \cos (\theta _1+\theta _2),\cos (\theta _1-\theta _2) \}, \end{aligned}$$

where $\mathcal {I}_-:=\textrm{diag}(-1,1,1,1)$, and $\theta _1$, $\theta _2$ denote the principal angles between $\mathcal {P}(x,y)$ and $\mathcal {P}(u,v)$.

Note that the right-hand side of (F.23) is $\pm \{\xi _+,\xi _-\}$ for $\xi _+,\xi _-$ as in (F.2).

#### Proof of Lemma F.6

The product measure $\sigma _{{\mathbb {S}}^2}\otimes \sigma _{{\mathbb {S}}^2}$ is $\textrm{SO}(3)\times \textrm{SO}(3)$ invariant. According to (F.17), the pushforward measure of $\sigma _{{\mathbb {S}}^2}\otimes \sigma _{{\mathbb {S}}^2}$ on ${\mathbb {S}}^2 \times {\mathbb {S}}^2$ under $\mathcal {P}$ is $\textrm{SO}(4)$ invariant, so that the uniqueness of the Haar measure implies the first claim of the lemma.

Each of the remaining claims is first proved for $\mathcal {P}$ as defined in Proposition F.4 and then argued that it also holds for $I_4-\mathcal {P}$. The identity (F.20) is easily observed for $u,v=e_1$ first and then (F.17) yields the general case. According to

$$\begin{aligned} \langle I_4-\mathcal {P}(x,y),I_4-\mathcal {P}(u,v)\rangle _{\textrm{F}} =\langle \mathcal {P}(x,y),\mathcal {P}(u,v)\rangle _{\textrm{F}}, \end{aligned}$$

the identity (F.20) also holds for $I_4-\mathcal {P}$. The statement (F.21) follows from expanding both sides of the equality and comparing polynomial expressions in *x* and *y*. If it holds for $\mathcal {P}$, then it must also hold for $I_4-\mathcal {P}$. One directly calculates (F.22).

In order to check (F.23), we first recognize that principal angles between $I-\mathcal {P}(x,y)$ and $I-\mathcal {P}(u,v)$ coincide with the ones between $\mathcal {P}(x,y)$ and $\mathcal {P}(u,v)$. By $\xi _{\pm }$ as in (F.2) and as at the beginning of the proof of Theorem 7.1, we observe $\textrm{trace}(\mathcal {P}(x,y)\mathcal {P}(u,v))=1+\xi _+\xi _-$. Hence, (F.20) leads to $\xi _+\xi _- = \langle x,u\rangle \langle y,v\rangle .$ Theorem 7.2 implies that $Q_\lambda$ in (7.2) satisfies

$$\begin{aligned} Q_\lambda \left( \mathcal {P}(x,y),\mathcal {P}(u,v)\right)= & {} \frac{c_\lambda }{2} \left( \mathcal {C}^{\frac{1}{2}}_{\lambda _1 +\lambda _2}(\langle x,u\rangle )\mathcal {C}^{\frac{1}{2}}_{\lambda _1-\lambda _2}(\langle y,v\rangle )\right. \\{} & {} \left. + \mathcal {C}^{\frac{1}{2}}_{\lambda _1+\lambda _2}(\langle y,v \rangle ) \mathcal {C}^{\frac{1}{2}}_{\lambda _1-\lambda _2}(\langle x,u \rangle )\right) . \end{aligned}$$

Comparison of this identity with (F.1) and few further calculations eventually lead to (F.23). $\square$

#### Proof of Theorem 7.2

The action of $\textrm{SO}(3)\times \textrm{SO}(3)$ leads to the irreducible decomposition

The unit quaternions provide a group structure on $\mathbb {S}^3$, so that the mapping $a\mapsto S_a$ between  and $\textrm{SO}(3)$ as well as $(a,b)\mapsto L_a R_b$ between  and $\textrm{SO}(4)$ become group isomorphisms. Their combination induces a group isomorphism between $\textrm{SO}(3)\times \textrm{SO}(3)$ and . Condition (F.17) requires that the respective actions on $\mathbb {S}^2\times \mathbb {S}^2$ and $\mathcal {G}_{2,4}$ commute with $\mathcal {P}$. Hence, the induced pullback

is an intertwining isomorphism. In particular, $\mathcal {P}^*$ maps one irreducible subspace into the other. Thus, the irreducible decomposition of $L_2(\mathcal {G}_{2,4})$ under the action of $\textrm{SO}(4)$ is

F.24 $$\begin{aligned} L_2(\mathcal {G}_{2,4}) =\bigoplus _{m+n\in 2\mathbb {N}} \textrm{span}\{ Y_{k,l}^{m,n}: k = -m,\dots ,m,\; l=-n,\ldots ,n\}. \end{aligned}$$

In comparison to the irreducible decomposition of $L_2(\mathcal {G}_{2,4})$ for the action of $\textrm{SO}(4)$ in (F.24), the irreducible components $H_\lambda (\mathcal {G}_{2,4})$ with respect to $\textrm{O}(4)$ in (7.1) are usually larger.

Let us denote $H^{m,n}:=\textrm{span}\{ Y_{k,l}^{m,n}: k = -m,\dots ,m,\; l=-n,\ldots ,n\}$. Property (F.21) yields that the irreducible subspaces of $L_2(\mathcal {G}_{2,4})$ under the action of $\textrm{O}(4)$ are

$$\begin{aligned} V^{m,n}:= {\left\{ \begin{array}{ll} H^{m,n} ,&{} m=n,\\ H^{m,n}\oplus H^{n,m},&{} m\ne n, \end{array}\right. } \end{aligned}$$

where $$m+n\in 2\mathbb {N}$$ and $$m\ge n$$, i.e.,

$$\begin{aligned} L_2(\mathcal {G}_{2,4}) = \bigoplus _{\begin{array}{c} m+n\in 2\mathbb {N}\\ m\ge n \end{array}} V^{m,n}=\bigoplus _{\lambda _1\ge \lambda _2\ge 0} V^{m_\lambda ,n_\lambda }, \end{aligned}$$

where $$m_\lambda =\lambda _1+\lambda _2$$ and $$n_\lambda =\lambda _1-\lambda _2$$.

By considering $$\mathcal {L}(P)=xy^\top $$, $$\mathcal {L}(P)\cdot \mathcal {L}(P)^\top =xx^\top $$, and $$\mathcal {L}(P)^\top \cdot \mathcal {L}(P)=yy^\top $$, we observe that the homogeneous polynomials $$ x_{i}y_{j}$$ and $$x_{i}x_{j}$$, $$y_{i}y_{j}$$, for $$i,j = 1,\dots ,3, $$ can be written as homogeneous polynomials in the matrix entries of $$P \in \mathcal {G}_{2,4}$$ of degree 1 and 2, respectively. The monomial $$x^\alpha y^{\beta }$$ with $$|\alpha |=m_\lambda $$ and $$|\beta |=n_\lambda $$ is composed of $$n_\lambda $$ factors of the form $$x_iy_j$$ and $$(m_\lambda -n_\lambda )/2$$ factors of the form $$x_{i}x_{j}$$. Thus, $$x^\alpha y^{\beta }$$ is a homogeneous polynomial of degree $$n_\lambda +2((m_\lambda -n_\lambda )/2) = m_\lambda $$ in the matrix entries of *P*. We deduce

F.25 $$\begin{aligned} \bigoplus _{\lambda _1+\lambda _2\le t} V^{m_\lambda ,n_\lambda } \subset \bigoplus _{\lambda _1+\lambda _2\le t} H_\lambda (\mathcal {G}_{k,d}) \end{aligned}$$

since the right-hand side of (F.25) are the polynomials of degree at most *t* in the matrix entries of $P\in \mathcal {G}_{2,4}$, cf. [8]. By applying [26, Formulas (24.29) and (24.41)], we see that the dimension of $H_{\lambda }(\mathcal {G}_{2,4})$ is

F.26 $$\begin{aligned} \textrm{dim}(H_{\lambda }(\mathcal {G}_{2,4})) =(2-\delta _{m_\lambda ,n_\lambda })(2m_\lambda +1)(2n_\lambda +1), \end{aligned}$$

which matches $\dim (V^{m_\lambda ,n_\lambda })$. Hence, there holds equality in (F.25). For fixed *t*, the dimensions in (F.26) are pairwise different, for all $\lambda _1+\lambda _2=t$. An induction over *t* leads to $H_{\lambda }(\mathcal {G}_{2,4})=V^{m_\lambda ,n_\lambda }$. $\square$

### F.3. Proofs for Section 7.3

The relation (7.16) yields

$$\begin{aligned} F(x,y) = \sum _{k,l=-M}^M e^{\textrm{i}k\varphi _1}e^{\textrm{i}l\varphi _2} B_{k,l}(\theta _1,\theta _2), \end{aligned}$$

where $B_{k,l}(\theta _1,\theta _2)$ are given by

$$\begin{aligned} B_{k,l}(\theta _1,\theta _2)&:= \sum _{m_1=|k|}^M\;\sum _{m_2=|l|}^M f^{m_1,m_2}_{k,l} \sum _{k'=-m_1}^{m_1} \sum _{l'=-m_2}^{m_2} c^{m_1}_{k,k'} c^{m_2}_{l,l'} e^{\textrm{i}k'\theta _1} e^{\textrm{i}l'\theta _2}\\&= \sum _{k',l'=-M}^M e^{\textrm{i}k'\theta _1}e^{\textrm{i}l'\theta _2} \sum _{m_1=\max (|k|,N-k')}^M \;\;\sum _{m_2=\max (|l|,M-l')}^M f^{m_1,m_2}_{k,l} c^{m_1}_{k,k'} c^{m_2}_{l,l'}. \end{aligned}$$

Hence, the coefficients from (7.17) satisfy

F.27 $$\begin{aligned} b^{k,l}_{k',l'} = \sum _{m_1=\max (|k|,M-k')}^M c^{m_1}_{k,k'} \sum _{m_2=\max (|l|,M-l')}^M f^{m_1,m_2}_{k,l} c^{m_2}_{l,l'}. \end{aligned}$$

First evaluating the inner sum for $k,l,l'=-M,\ldots ,M$ and $m_1=\max (|k|,M-k'),\ldots ,M$, and afterwards the outer sum enables the computation of the coefficients $b^{k,l}_{k',l'}$, for $k,l,k',l'=-M,\ldots ,M$, in $O(M^5)$ operations provided that the numbers $c^{m}_{k,k'}$ in (7.16) are given.

#### Remark F.7

The complexity for evaluating the two sums in (F.27) can be further reduced to $O(M^4\log ^2(M))$ by using a fast polynomial transform, cf. [39, 41]. In this way, the nonequispaced fast Fourier transform on $\mathbb {S}^2\times \mathbb {S}^2$ takes $O(M^4\log ^2(M)+n|\log (\epsilon )|^4)$ elementary operations. For $n\sim M^4$, this implies the reduction of complexity from $O(M^8)$ to $O(M^4\log ^2(M)+M^4|\log (\epsilon )|^4)$ operations.

The adjoint nonequispaced fast Fourier transform is about computing

F.28 $$\begin{aligned}{} & {} \sum _{i=1}^M b_i \overline{Y^m_k(x_i)Y^n_l(y_i)},\nonumber \\{} & {} \quad m,n=0,\ldots ,N,\;\; k=-m,\ldots ,m,\;\; l=-n,\ldots ,n, \end{aligned}$$

for given coefficients $(b_i)_{i=1}^M\subset \mathbb {C}$ and locations $(x_i,y_i)_{i=1}^M\subset \mathbb {S}^2\times \mathbb {S}^2$. Similar arguments as above and in Sect. 7.3 provide a fast evaluation of (F.28) by using the adjoint nonequispaced fast Fourier transform adjoint nfft, cf. [39, 41].

## Footnotes

1 If $\beta$ is a pushforward measure, then the associated sigma algebra on $\mathscr {B}(\mathbb {R}^d)$ is induced by the pushforward.

2 For $r\in \mathbb {R}$, we use the notation $$r_+={\left\{ \begin{array}{ll} r,&{} r\ge 0,\\ 0,&{} \text {otherwise.} \end{array}\right. }$$

3 The kernel $K_{\beta _d}|_{\mathbb {S}^{d-1}\times \mathbb {S}^{d-1}}$ generates an inner product $\langle \cdot ,\cdot \rangle _{K_{\beta _d}|_{\mathbb {S}^{d-1}\times \mathbb {S}^{d-1}}}$ in $\mathbb {H}^{\frac{d}{2}}(\mathbb {S}^{d-1})$, for which it is reproducing and the induced norm is equivalent to the standard norm in $\mathbb {H}^{\frac{d}{2}}(\mathbb {S}^{d-1})$, which is induced by the standard kernel (3.3).

4 The geodesic distance on $\mathbb {S}^2$ induces the geodesic distance on  by
